# Enhanced geometry control powered by AI for UAVS with a robotic arm for compensating for disturbances

**DOI:** 10.1038/s41598-026-52048-y

**Published:** 2026-05-22

**Authors:** Khaled Oqda, Eman M. El-Gendy, Hanaa Salem Marie, Mohamed Akalla

**Affiliations:** 1 Mechatronics Engineering Department, Faculty of Engineering, Mansoura University, Mansoura, Egypt; 2 Computers and Control Systems Engineering Department, Faculty of Engineering, Mansoura University, Mansoura, Egypt; 3 Faculty of Artificial Intelligence, Delta University for Science and Technology, Gamasa, 35712 Egypt; 4 Mechatronics and Robotics Eng. Dept, University of Science and Technology (E-JUST), Alexandria, 21934 Egypt; 5 Production Engineering and Mechanical Design Dept, Faculty of Engineering, Mansoura University, 35516 Mansoura , Egypt

**Keywords:** UAV, Robotic Arm, Geometric Control, SO(3), LSTM, Disturbance Compensation, Real-time Systems, Distributed Computing, Engineering, Mathematics and computing

## Abstract

Unmanned Aerial Vehicles (UAVs) equipped with robotic manipulators have emerged as a powerful paradigm for advanced aerial manipulation tasks, including infrastructure inspection, emergency response, and precise object handling in hazardous or inaccessible environments. Despite their potential, integrating robotic arms introduces significant challenges due to strong dynamic coupling, time-varying payloads, and external disturbances such as wind and aerodynamic turbulence, which can severely compromise flight stability and control performance. Conventional linear and nonlinear contrDistributed Control Architecture strategies often struggle to cope with these highly nonlinear dynamics, particularly during aggressive manipulator motions. This paper proposes an AI-enhanced geometric control framework for stabilising UAV–manipulator systems under high-manoeuvrability conditions. By operating directly on the nonlinear configuration space, geometric control provides robust attitude and position stabilization while avoiding singularities and ensuring global stability. To overcome the limited adaptability of purely geometric controllers, artificial intelligence algorithms are integrated to predict and compensate for manipulator-induced disturbances, optimize thrust distribution, and dynamically adjust BLDC motor currents in real time. The integration of robotic manipulators with Unmanned Aerial Vehicles (UAVs) offers a transformative approach to aerial manipulation in hazardous environments. However, the shifting center of mass and aerodynamic coupling during arm movement present significant stabilization challenges. This paper proposes a hybrid control framework that combines Geometric Control on the SO(3) manifold with a Long Short-Term Memory (LSTM) neural network. The LSTM is specifically designed to predict and compensate for non-linear disturbances and dynamic coupling effects in real-time. Experimental results demonstrate that this AI-enhanced geometric approach provides superior attitude tracking and disturbance rejection compared to traditional PID and sliding mode control, significantly reducing oscillation during complex maneuvers. A quadrotor UAV equipped with a Cartesian robotic arm employs the proposed framework for compensation of disturbances affecting both position and orientation. Simulation results obtained using a high-fidelity CoppeliaSim–MATLAB environment demonstrate the effectiveness of the proposed approach. The UAV maintains stable flight and accurate attitude regulation while carrying relatively high payloads and executing rapid robotic arm maneuvers.

## Introduction

The integration of robotic manipulators with Unmanned Aerial Vehicles (UAVs) represents a significant advancement in the evolution of autonomous aerial systems. Over the past decade, UAVs have evolved from simple platforms used mainly for photography and reconnaissance into capable robotic agents able to physically interact with their environment^1^. Equipping UAVs with robotic arms greatly expands their operational scope, enabling applications such as precision object manipulation, emergency response in hazardous environments, infrastructure inspection and maintenance, sample collection in remote areas, and disaster relief operations. As a result, aerial manipulation has emerged as one of the most promising research directions in robotics and mechatronics.Despite these advantages, this integration substantially increases system complexity. UAVs are inherently unstable platforms, and the addition of a robotic arm introduces significant disturbances that complicate stable flight control. Manipulator motion causes abrupt shifts in the center of mass, generates interaction forces, and produces nonlinear torques that can destabilize the UAV. These effects are further intensified by external disturbances such as wind, turbulence, and payload variability. Consequently, advanced and intelligent control strategies are required to anticipate disturbances, mitigate dynamic coupling, and ensure real-time adaptation^2^.

Early efforts to mount robotic arms on UAVs were constrained by limited computational power, sensing accuracy, and control methodologies. Traditional control approaches based on PID controllers, linearized models, or simplified UAV–manipulator representations provided acceptable performance for basic flight stabilization but failed to address the strong nonlinearities induced by manipulator motion. During high-speed arm movements or dynamic payload handling, these controllers often resulted in severe oscillations or loss of stability^3^. To address these challenges, researchers explored more advanced techniques such as adaptive control, backstepping, and nonlinear state-space modeling. Although these approaches improved performance in certain scenarios, they were often limited by high computational cost and difficulties in real-time implementation on lightweight UAV hardware. Moreover, external disturbances such as wind gusts and aerodynamic uncertainties continued to degrade system performance. Despite notable progress, achieving a control strategy that is simultaneously fast, robust, computationally efficient, and capable of handling full UAV–manipulator nonlinear dynamics remains a major challenge^4^.

Recently, geometric control has gained attention as a promising framework for UAV stabilization. By operating directly on nonlinear manifolds such a SO(3). It is a geometric controllers avoid singularities and offer strong theoretical stability guarantees. However, purely geometric approaches often lack adaptability when confronted with unpredictable disturbances arising from manipulator dynamics, payload changes, or environmental effects. Without additional learning or adaptation mechanisms, their effectiveness in real-world conditions can be limited^5^. In parallel, artificial intelligence (AI) techniques have shown success in UAV navigation and planning, yet their application in enhancing low-level UAV–manipulator control remains limited. Few studies have combined geometric stability theory with AI-driven prediction and adaptation^6^.

This research addresses these gaps by proposing a hybrid control framework that integrates geometric control with AI-based optimization and adaptation. The UAV platform considered in this study is a quadrotor equipped with a 3-DOF robotic arm (HANA-26), designed to emulate realistic aerial manipulation behavior. The system undergoes continuous variations in center-of-mass position, inertia, and torque disturbances due to arm motion, making it a suitable testbed for advanced control evaluation. A high-fidelity simulation environment is developed using CoppeliaSim, while MATLAB serves as the main control and data-processing platform^7^. Through a Remote API interface, MATLAB exchanges control signals and sensor feedback with the simulator, enabling real-time monitoring of flight stability using Euler-angle plots. This framework allows direct comparison between conventional PID control, standard geometric control, and the proposed AI-enhanced geometric controller^8^. The core contribution of this work is the development of an AI-enhanced geometric control framework that improves stability, responsiveness, and robustness of UAVs carrying robotic arms. The proposed controller combines geometric control for attitude and position stabilization with AI algorithms that optimize thrust distribution, adjust BLDC motor currents, and compensate for nonlinear disturbances in real time using sensor feedback^9,10^. Recent advancements in hybrid control have shown that AI can significantly enhance the disturbance rejection capabilities of traditional controllers. For instance^11^, demonstrates the effectiveness of adaptive strategies in maintaining stability under system faults. Similarly^12^, highlights the role of intelligent computation in modeling the complex interactions of UAV-manipulator systems. Our proposed LSTM-enhanced geometric controller builds on these insights, offering a more precise temporal prediction of disturbance torques compared to traditional adaptive observers.

Recent advancements in robotic control have introduced robust frameworks such as Adaptive Neural Network-based Fixed-Time Control, which ensures trajectory tracking convergence within a predefined time regardless of initial conditions^13^. Such methods are particularly effective for robots facing input saturation and those requiring prescribed performance to maintain safety margins during complex manipulation tasks^14^. By incorporating these nonlinear strategies, researchers have significantly improved the transient response and steady-state accuracy of multi-degree-of-freedom systems.

The main contributions of this research include the development of a detailed nonlinear dynamic model, formulation of a robust geometric control law, integration of AI-based adaptation, and comprehensive validation through CoppeliaSim–MATLAB simulations. The results demonstrate reduced oscillations, faster stabilization, improved disturbance rejection, and enhanced reliability during aggressive manipulator motion. The remainder of this paper is organized as follows. Section II presents the proposed UAV–manipulator system and its nonlinear dynamic model. Section III describes the control framework, including PID, geometric control, and the proposed AI-enhanced geometric controller. Section IV outlines the simulation setup, while Section V presents the results and discussion. Finally, Section VI concludes the paper and outlines future research directions.

## Developed UAV with robotic arm (HANA – 26)

### Mechanical design

**Fig. 1:**
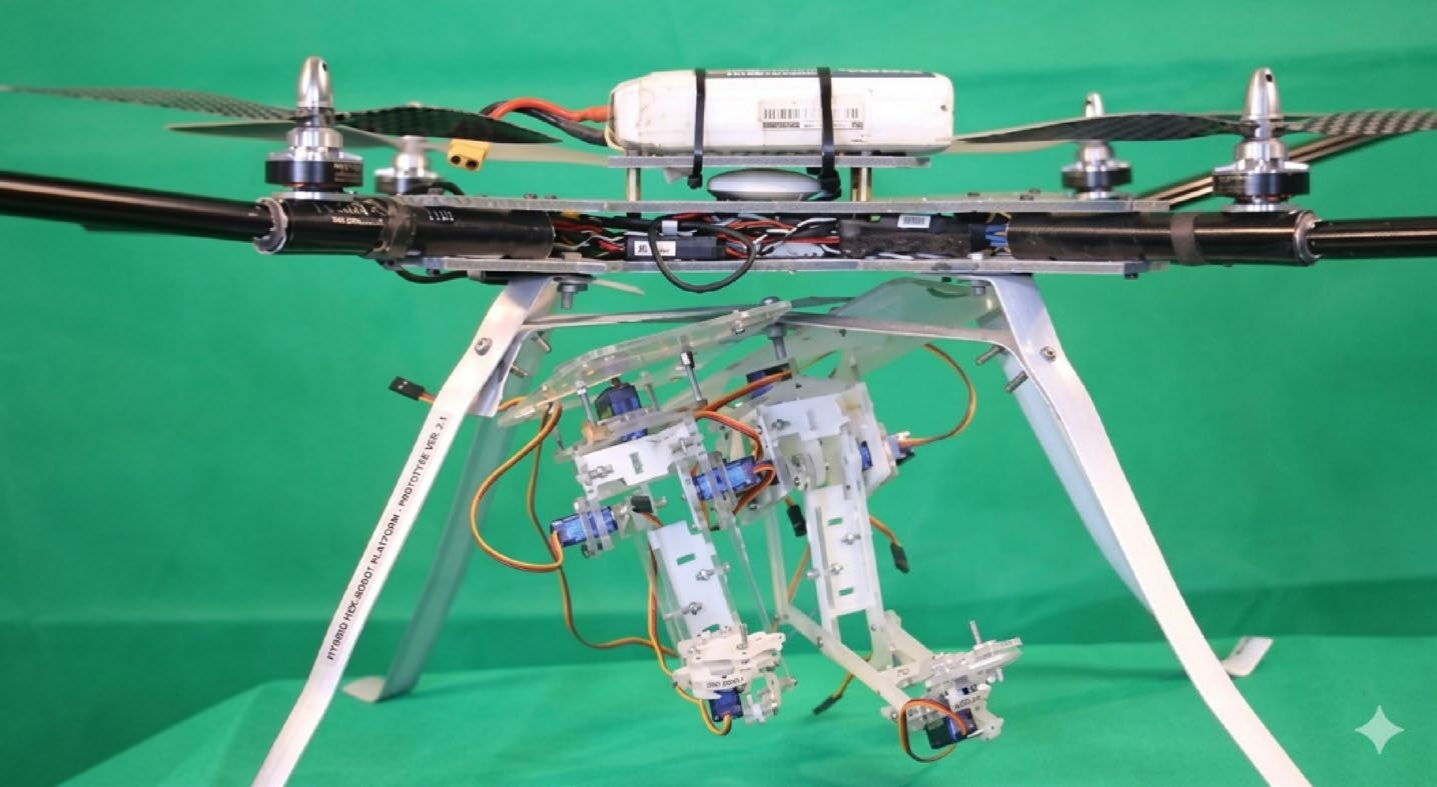
Original photograph of the experimental UAV-manipulator platform (HANA-26).

The proposed UAV system, designated as HANA-26, has been meticulously developed to ensure a lightweight, highly rigid, and structurally modular platform capable of supporting a fully actuated robotic arm without compromising flight stability or control precision as shown in Fig. 1. The fundamental philosophy of the design relies on maximizing structural strength while minimizing overall mass, which is achieved through the extensive use of advanced carbon-fiber composite materials. These composites provide an exceptional strength-to-weight ratio, grant high resistance to deformation, and significantly enhance the drone’s capacity to maintain stability under dynamic loading conditions generated by the movement of the robotic arm^15^. The mechanical architecture and structural components of the UAV and the 3-DOF robotic arm were developed and analyzed using SolidWorks 2023 and AutoCAD 2023.

**Fig. 2:**
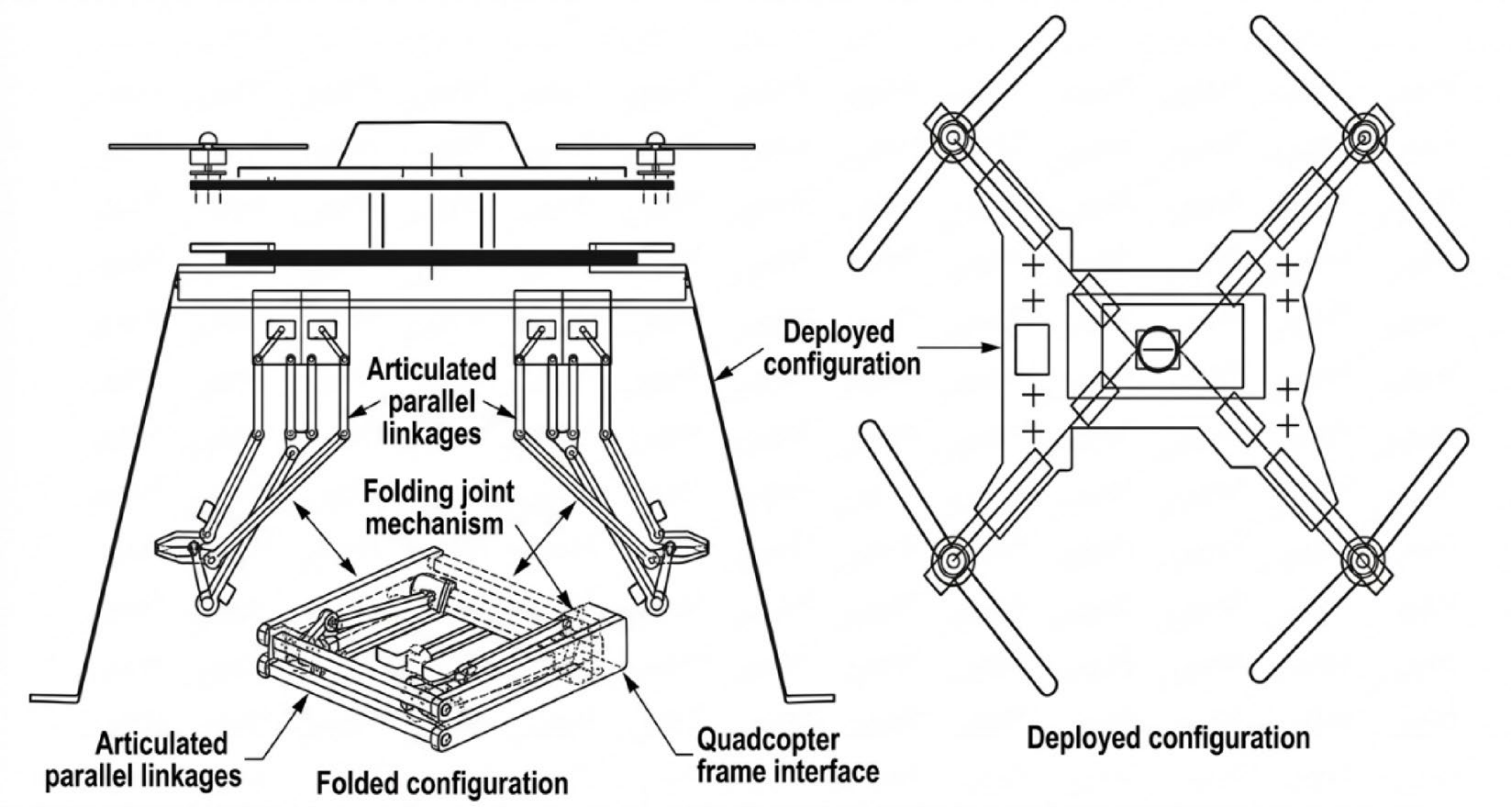
Structural layout and dimensional analysis of the robotic arm (designed using AutoCAD 2023).

To ensure optimal balance, the 3-DOF robotic arm is mounted precisely at the center of mass (CoM) of the UAV. Positioning the arm at this central location minimizes destabilizing moments and reduces the coupling effects that typically arise between arm motion and UAV attitude. Each joint of the arm employs precision, low-friction bearings, enhancing smooth rotational motion and ensuring minimal mechanical resistance during manipulation tasks. The mechanical configuration also considers the arm’s maximum reach, payload capacity, and the dynamic influence of rapid arm maneuvers on the stability of the quadrotor as shown in Fig. 2. These considerations collectively provide a resilient structure capable of standing up to operational stresses without exceeding tolerable deformation limits^16^.

**Fig. 3:**
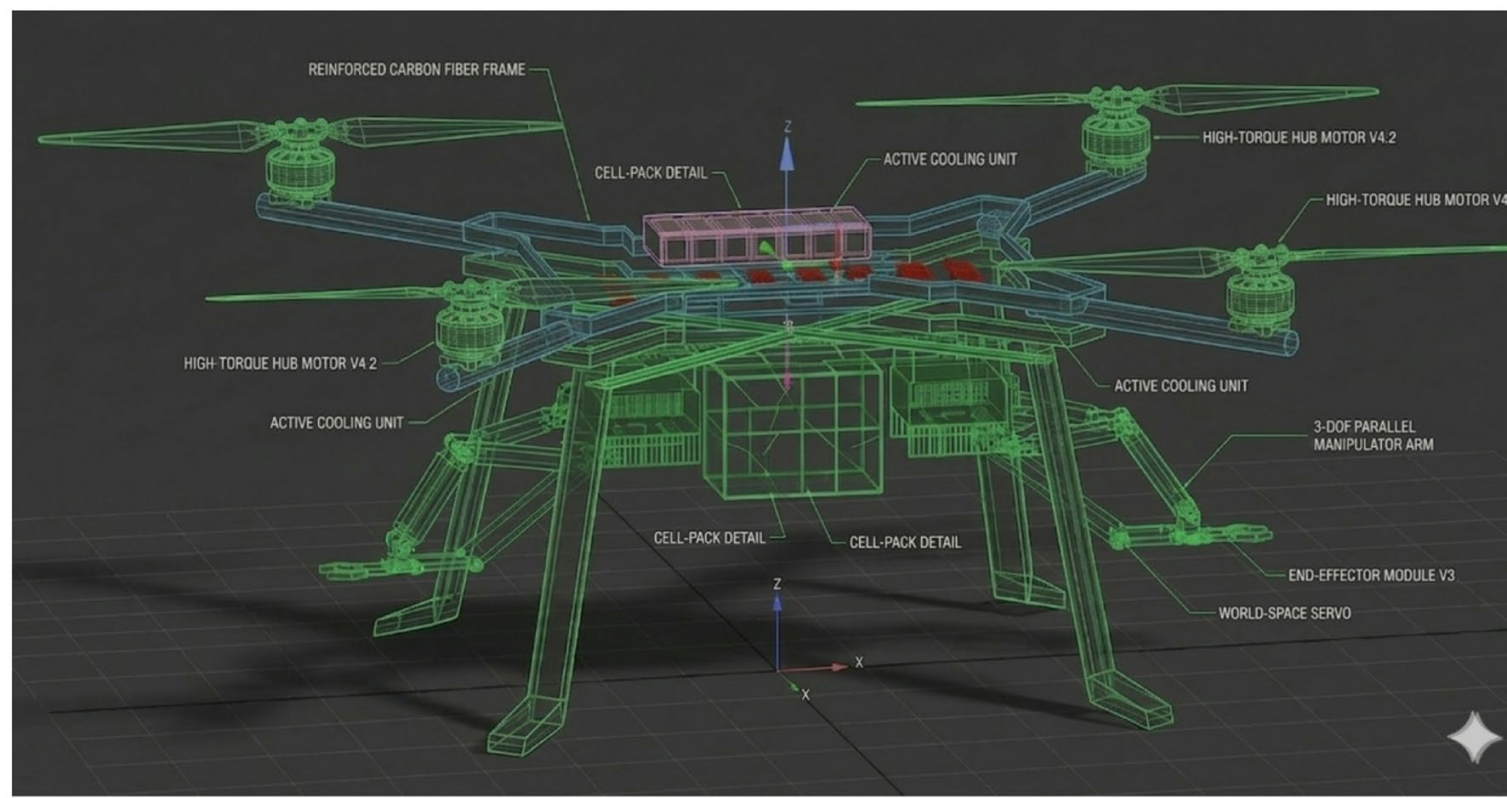
UAV mechanical design and 3D assembly model (generated using SolidWorks 2023).

The complete system is modeled using SolidWorks and full CAD assembly simulation, allowing precise mass-distribution control, mechanical interference inspection, and aerodynamic assessment. The drone’s outer shell adopts a semi-cylindrical aerodynamic profile that tapers forward, enclosing an internal electronic compartment while maintaining large, structurally strong extensions at the four corners for motor mounting^17^. These extensions stabilize thrust vectors, support vertical and horizontal motors, and preserve the overall momentum direction of the UAV during flight as shown in Fig. 3.

The mechanical structure is further reinforced by integrated metallic support links surrounding the carbon-fiber frame. These links improve rigidity and ensure the preservation of the UAV’s center of balance during dynamic transitions such as take-off, landing, and aerial manipulation. The leg system of HANA-26 consists of four articulated aluminum legs, combining fixed and moving mechanical joints to absorb impact during landings and protect the robotic arm and body from structural shocks. Aluminum is chosen due to its superior flexibility, light mass, and resistance to fatigue^18^. From an aerodynamic perspective, the UAV is designed with neutral buoyancy characteristics, balancing internal air volume with the lightweight carbon-fiber shell. This enhances stability and improves flight duration^19^. A detailed geometric layout illustrates the placement of the unsprung motors at the branch ends, facilitating efficient vertical thrust generation. The mechanical design reflects the integration of aerodynamic efficiency, structural integrity, and protective coverage against external environmental influences such as wind, dust, rain, and mechanical vibrations. Propulsion Modeling and Propeller Dynamics The propeller behavior is characterized using classical aerodynamic parameters: the thrust coefficient (cT), power coefficient (cP), and propeller radius (r). The rotor’s mechanical power output is expressed as:

1 $$\:F={C}_{T}\frac{4{\rho\:r}^{4}}{{\pi\:}^{2}}{\varOmega\:}^{2}$$

2 $$\:P={C}_{P}\frac{4{\rho\:r}^{5}}{{\pi\:}^{3}}{\varOmega\:}^{3}$$

**Fig. 4:**
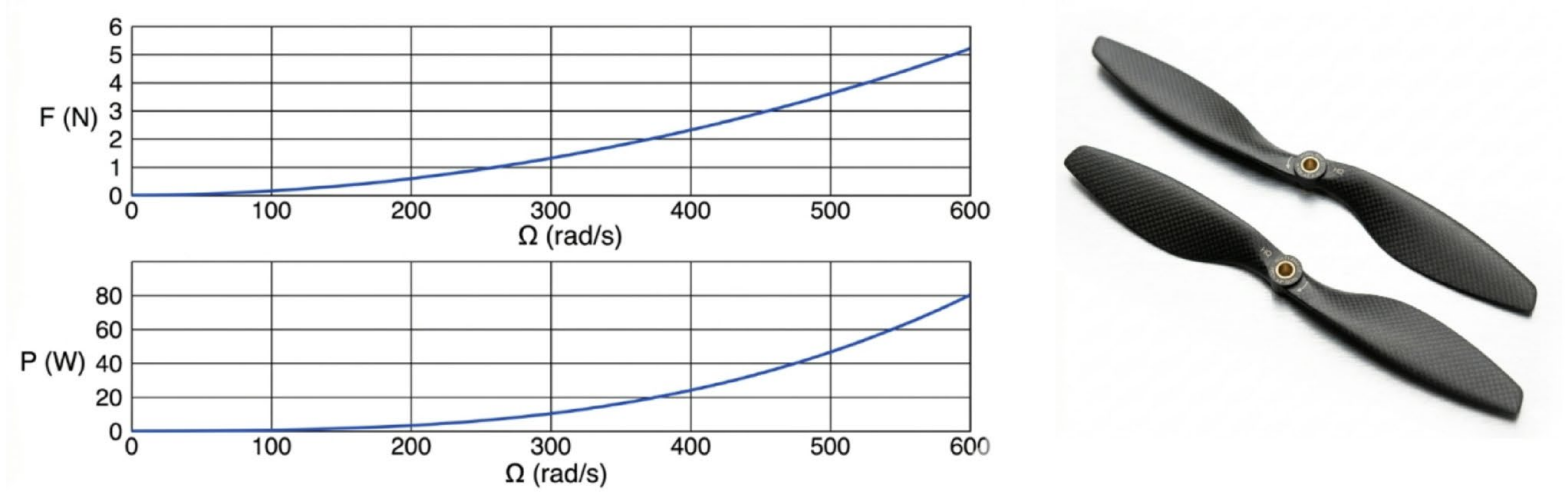
Theoretical thrust and power of an EPP1045 Propeller^22^.

These relationships demonstrate how propeller radius, aerodynamic drag, and angular velocity influence total thrust and power consumption. A larger propeller diameter delivers higher thrust at lower speeds, enhancing flight autonomy by reducing motor power demands. The chosen EPP1045 propeller (10-inch diameter, 23 g weight) offers the optimal balance between thrust, aerodynamic efficiency, and structural durability. For lift-off, each propeller must generate at least 375 g of thrust at approximately 490 rad/s, requiring roughly 43 W of power, fulfilling the system’s weight support requirements as shown in Fig. 4^20^.

### Electrical system architecture

The electrical subsystem of HANA-26 is engineered to provide stable, efficient, and reliable power distribution for both the quadrotor and the robotic arm. Each rotor is driven by a high-efficiency Brushless DC (BLDC) motor controlled by Electronic Speed Controllers (ESCs) capable of precise current modulation. A 6 S Li-Po battery (5000 mAh) supplies the UAV with high-discharge, lightweight power suitable for both long-duration flight and dynamic robotic manipulation tasks. The propulsion system comprising motors, ESCs, batteries, and propellers plays a central role in determining thrust capacity, hovering time, and maneuverability. HANA-26 employs outer-rotor BLDC motors, known for providing higher torque and smoother rotational dynamics, improving stability under heavy payloads. Given the system’s maximum weight (1.5 kg), each rotor must provide a sTable 700 g of lift to ensure adequate airborne performance, especially when carrying the arm’s maximum payload of 0.9 kg. as shown in Table 1.

**Table 1:** Components and parameter values.

Weight	2.8 kg	Max reach	30 cm
Dimensions	600 mm x 600 mm x 400 mm	Max payload	250
Battery Lippo	8–12 S LiPo	type	Brushless Outrunner
Max Air speed	20 m/s	Model	MAD Shadow XC3000 T2 V1.0
Max thrust	64 N	KV	800 KV
Servo wight	50 g	Voltage	8–12 S LiPo
Max torqe/1 motor	3.2 kg.cm	max current	28–30 A
Max torqe/2 motor	0.6278 Nm	ALL max thrust	≈ 0.9 Kg
Arm wight	350gm	Brushless motor wight	≈ 70 gm

### Electronic, sensor, and control hardware

The electronic architecture integrates all sensing, computing, and communication modules into a cohesive real-time control system. Inertial Measurement Unit (IMU) used for estimating roll, pitch, and yaw angles, providing high-frequency attitude feedback for geometric control, Force Sensors and Joint Encoders, mounted on the robotic arm to measure interaction forces, joint positions, and joint velocities critical for stable manipulation and adaptive control, Flight Controller + Dual PIC18F Microcontrollers.The UAV employs a hybrid control structure consist of The flight controller, which executes geometric control laws and communicates with MATLAB through the Remote API, Two embedded PIC18F microcontrollers, programmed in C++, to handle the robotic arm motion, servo feedback filtering, and force sensor processing, MATLAB commands, and CoppeliaSim feedback are exchanged rapidly, enabling effective real-time state estimation and adaptive AI-enhanced geometric control^21^.

### Dynamic model for proposed system

Euler Angles are used to represent the quadrotor’s rotational mechanics. The position and orientation of a rigid body with respect to a reference frame, such as X Y Z, which is assumed to be earth-fixed and inertial. The rotation R matrix needed to transform the linear velocities is expressed in terms of Euler angles by the following matrix:

3 $$R=\:\left[\begin{array}{ccc}C\left(\psi\:\right)C\left(\theta\:\right)&\:S\left(\psi\:\right)C\theta\:&\:\:-S\left(\theta\:\right)\\\:-S\left(\psi\:\right)C\left(\varphi\:\right)+C\left(\psi\:\right)S\left(\theta\:\right)S\left(\varphi\:\right)&\:C\left(\psi\:\right)S\left(\varphi\:\right)+S\left(\psi\:\right)S\left(\theta\:\right)C\left(\varphi\:\right)&\:C\left(\theta\:\right)S\left(\varphi\:\right)\\\:S\left(\psi\:\right)S\left(\varphi\:\right)+C\left(\psi\:\right)S\left(\theta\:\right)C\left(\varphi\:\right)\:\:\:&\:-C\left(\psi\:\right)S\left(\varphi\:\right)+S\left(\psi\:\right)S\left(\theta\:\right)C\left(\varphi\:\right)&\:\:C\left(\theta\:\right)C\left(\varphi\:\right)\end{array}\right]$$

where C() and S() are short notations for cos() and sin().

the rotor j’s angular velocity, represented by Wj, produces a drag moment Mj and a thrust force Fj. The thrust force and drag moment are both proportional to the square of the propeller’s angular speed, according to the momentum theory. According to the following, the power of the rotor j, Pj, is equal to the drag moment times the angular velocity. The formula is represented by Matrix (J_v_).

4 $${\mathrm{J}}_{{\mathrm{v}}} = \left[ {\begin{array}{*{20}c} 1 & 0 & {{\mathrm{~}} - {\mathrm{S}}\left( {{\theta }} \right)} \\ 0 & {{\mathrm{C}}\left( {{\varphi }} \right)} & {{\mathrm{C}}\left( {{\theta }} \right){\mathrm{S}}\left( {{\varphi }} \right)} \\ 0 & { - {\mathrm{S}}\left( {{\varphi }} \right)} & {C\left( \theta \right)C\left( \varphi \right)} \\ \end{array} } \right]~$$

The thrust force and drag moment are both proportional to the square of the propeller’s angular speed, according to the momentum theory

5 $$\:{F}_{j}={K}_{Fj}{{\varOmega\:}_{j}}^{2}\:\:\:\:\:\:\:$$

6 $$\:{M}_{j}={K}_{Mj}{{\varOmega\:}_{j}}^{2}\:\:\:\:\:\:$$

where the constants K_Mj_ and K_Fj_, respectively, relate the thrust and propeller moment to the angular speed^22^.

The Newton-Euler formalism is used to determine the quadrotor’s equation of motion as shown:

7 $$\:m\ddot{x}=T(C\left(\varPsi\:\right)S\left(\theta\:\right)C\left(\varphi\:\right)+S\left(\varPsi\:\right)S\left(\varphi\:\right)\:\:\:\:\:\:\:\:\:\:\:\:$$

8 $$\:m\ddot{Y}=T(C\left(\varPsi\:\right)S\left(\theta\:\right)C\left(\varphi\:\right)-S\left(\varPsi\:\right)S\left(\varphi\:\right)\:\:\:\:\:\:\:\:\:\:\:\:$$

9 $$\:m\ddot{Z}=-mg+T(C\left(\theta\:\right)C\left(\varphi\:\right)\:\:\:\:\:\:\:\:\:\:\:\:$$

10 $$\:I_{X} \mathop {\varphi \:}\limits^{{..}} = \mathop {\theta \:}\limits^{.} \mathop {\varphi \:}\limits^{.} \:\left( {I_{Y} - I_{Z} } \right) - \:\mathop {\theta \:}\limits^{.} \bar{\Omega }\:I_{r} + T$$

11 $$I_{y} \mathop {\theta \:}\limits^{{..}} = \mathop {\psi \:}\limits^{.} \mathop {\varphi \:}\limits^{.} \:\left( {I_{z} - I_{x} } \right) - \:\mathop {\varphi \:}\limits^{.} \:\bar{\Omega }\:I_{r} + T_{{a2}}$$

12 $$\:I_{z} \mathop {\psi \:}\limits^{{..}} = \mathop {\theta \:}\limits^{.} \mathop {\varphi \:}\limits^{.} \:\left( {I_{x} - I_{y} } \right)\: + T_{{a3}} \:$$

Assuming that the three angles f, q, and y vary slightly, the final three equations are obtained such that the appropriate time derivatives of Euler angles equal the body-fixed angular velocities, or J_v_ = I(3 × 3), so that Eq. (12) becomes, The following definitions apply to the variables in the equations above:

13 $$\:{\nu\:}_{2}={\dot{\eta\:}}_{2}\:\:\:\:\:\:\:\:\:\:\:\:\:\:\:$$

14 $$\:T=\sum\:_{J=1}^{4}\left({F}_{j}\right)\:\:\sum\:_{j=1}^{4}{K}_{Fj}\left({{\varOmega\:}^{2}}_{J}\right)$$

15 $${\mathrm{T}}_{{a1}} = d\left( {F_{4} F_{2} } \right)\:$$

16 $$\:{\mathrm{T}}_{a2}=d\left({F}_{1}{F}_{3}\right)\:\:\:\:\:\:\:\:\:\:\:\:\:\:\:\:$$

17 $$\:{\mathrm{T}}_{a3}={M}_{1}+{M}_{2}-{M}_{3}+{M}_{4}\:\:\:\:\:\:\:$$

18 $$\:\stackrel{-}{\varOmega\:}\:={\varOmega\:}_{1}+{\varOmega\:}_{2}+{\varOmega\:}_{3}+{\varOmega\:}_{4}\:\:\:\:\:\:\:\:\:\:\:\:$$

19 $${\text{W }} = {\text{ W1 }} - {\text{ W2 }} + {\text{ W3 }} - {\text{ W4}}$$

^23^

where the quadrotor’s mass is denoted by m. T is the total thrust that all four rotors provide to the quadrotor: Total Thrust and _a1_, _a2_, and _a3_ are the three input moments about the three body axes, These moments are the rolling, pitching, yawing moment about x, y, and z-axis of the body frame respectively.

Rotor inertia is denoted by Ir. Assuming that the vehicle is symmetric around the x, y, and z axes, the inertia matrix of the vehicle around its body frame is given by:

20 $$if=\:\left[\begin{array}{ccc}\:Ix&\:0&\:0\\\:0&\:Iy&\:0\\\:0&\:0&\:Iz\end{array}\right]$$

All of the quadrotor’s components are modeled using SOLIDWORKS to create a CAD model, as seen in Fig. 2. Motors, electronic components, batteries, and the aluminium frames that are put together are the components that are modeled. The arms of the rotor are made from a 1 mm-thick aluminium sheet. These arms are etched to reduce the overall weight and aerodynamic resistance while in flight. Using the principal laws to calculate the mass moment of inertia is made more difficult by all of these holes. Thus, the CAD model is used to directly extract the mass moments of inertia of the rotors and quadrotor framework. The CAD model yielded a diagonal, positive-definite inertia matrix. The mass moment of inertia about the body frame’s x, y, and z axes, the total mass, the rotor’s mass moment of inertia (I_r_), and the centre distance (d) between the rotor Axis and the center of the quadrotor.

### Dynamic model for Robotic Arm

The base of the Arm and its succeeding joints were given coordinate frames. The forward kinematic (FK) solution of the manipulator was obtained using the extremely precise Denavit-Hartenberg (DH) approach. The individual homogeneous transformation matrices between manipulator connections are computed using this method. The position of the end-effector in relation to the robot’s base is then described by the total homogeneous transformation matrix, which is obtained by successively multiplying each matrix. This technique describes the relative position and orientation of links in a manipulator chain using four parameters, which are as follows:

1. a_i_ – Link length: Distance between frame centres along X_i_.
2. α_i_ – Link twist: Angle between Z_i_ and Z_i+1_ about X_i_.
3. d_i_ – Link offset: Distance between frame centres along Z_i_.
4. θ_i_ – Joint angle: Angle between X_i-1_ and X_i_ about Z_i_.

The Arm DH parameters were located and entered into Table 2. Since there were four coordinate frames in the manipulator’s analytical model, the homogeneous transform for each frame was determined separately and then multiplied successively.

The complete dynamic model of the uArm Swift Pro robotic arm using the Lagrangian method. The model includes kinetic and potential energy calculations, the derivation of the Lagrangian, and the final dynamic equations in the form of Eq. 21.

21 $$\:{M}_{q}\ddot{q}+{C}_{(q,\ddot{q)}}\dot{q+}{G}_{\left(q\right)}=\tau\:$$

Denavit-Hartenberg Parameters Estimated in Table 2.

**Table 2:** Denavit-Hartenberg Parameters.

Joint	Θi	αi (deg)	a.i. (mm)	di (mm)
1	θ1	90	13.2	106.1
2	θ2	0	142	0
3	θ3	0	158.8	0
4	θ4	0	44.5	0

Link Mass Distribution of The total mass of the arm is 0.35 kg, and it is approximately distributed as follows:.

- Link 1: 0.01 kg - Link 2: 0.14 kg - Link 3: 0.16 kg - Link 4: 0.04 kg.

**Fig. 5:**
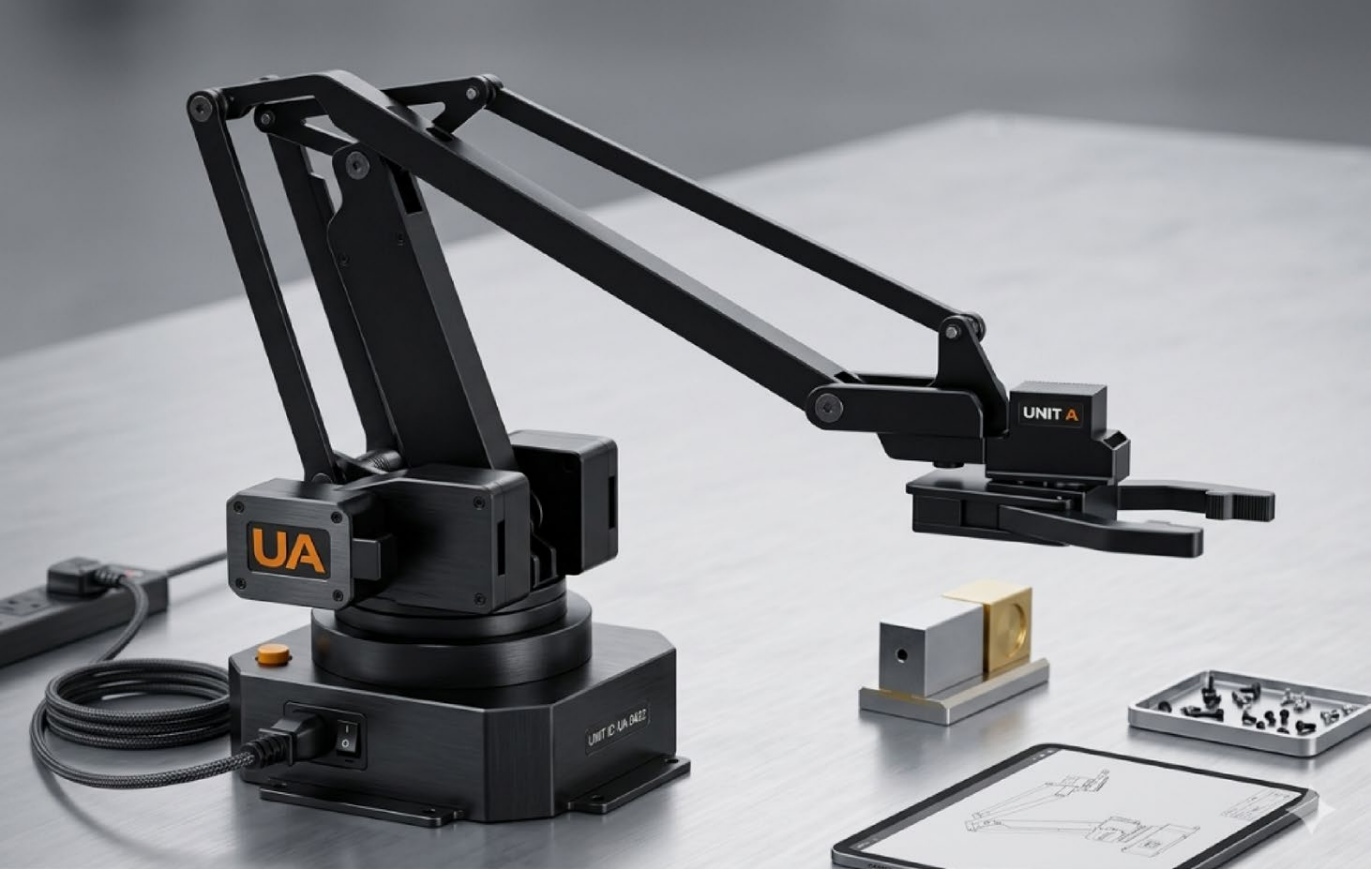
Detailed CAD visualization of the 3-DOF manipulator components (rendered in SolidWorks 2023).

The quad rotor UAV consists of four rotors with a maximum angular velocity of 4000 rpm and a maximum load capacity of 4 kg. The vehicle’s propulsion system, which consists of the four rotors, has a maximum load capacity of 6 kg. To model Carbon fiber is used for these purposes since it is a high-strength material with a total mass of 4 (kg). Propylene polymer, which is lightweight and extremely durable, is used to make the manipulation arm as shown in Fig. 5. This section displays a kinematic model of a quad rotor unmanned aerial vehicle with a manipulator arm to identify the position of the manipulator’s end-effector.

In order to identify the Euclidean spaces, the quadrotor UAV has six degrees of freedom (DOFs): three for orientation or attitude motion and three more for translation motion. explains the quadrotor UAV’s roll, pitch, and yaw movements. The red rotors on the positive X-axis represent the front of the quadrotor UAV. The green rotors on the positive Y-axis represent the right side of the multirotor when viewed from above. According to the right-hand rule, the positive Z-axis indicates a downward motion. the manipulator arm’s three degrees of freedom (DOFs) in a manner that maintains it firmly at the aerial vehicle’s bottom. This posture is considered for simulation analysis in Matlab when the arm is retracted and the octorotor UAV lifts off^23^.

22 $${\mathrm{R}}_{{\mathrm{B}}} ^{{\mathrm{1}}} = \:\left[\begin{array}{ccc}\mathrm{cos}\theta\:\mathrm{cos}\psi\:&\:\mathrm{sin}\varphi\:\mathrm{sin}\theta\:\mathrm{cos}\psi\:-\mathrm{cos}\varphi\:\mathrm{sin}\psi\:&\:\mathrm{cos}\varphi\:\mathrm{sin}\theta\:\mathrm{cos}\psi\:+\mathrm{sin}\varphi\:\mathrm{sin}\psi\:\\\:\mathrm{cos}\theta\:\mathrm{sin}\psi\:&\:\mathrm{sin}\varphi\:\mathrm{sin}\theta\:\mathrm{sin}\psi\:+\mathrm{cos}\varphi\:\mathrm{cos}\psi\:&\:\mathrm{cos}\varphi\:\mathrm{sin}\theta\:\mathrm{sin}\psi\:-\mathrm{sin}\varphi\:\mathrm{cos}\psi\:\\\:-\mathrm{sin}\theta\:&\:\mathrm{sin}\varphi\:\mathrm{cos}\theta\:&\:\mathrm{cos}\varphi\:\mathrm{cos}\theta\:\end{array}\right]\:\:\:\:\:\:\:\:\:\:\:\:\:\:$$

23 $$TB1=\:\left[\begin{array}{cc}\mathrm{R}\mathrm{B}1\:&\:\xi\:\\\:0&\:1\end{array}\right]$$

The matrix link between the UAV and the manipulator arm is given by a homogeneous matrix that describes the attitude and translation of the systems^24^. The rotation matrix (1) and the position vector ξ are taken into consideration while calculating the homogeneous matrix of the quad rotor UAV. Within matrix (2), the quadrotor UAV attitude’s Euler angles are (a) roll (φ), (b) pitch (θ), and (c) yaw (ψ). The kinematic model of the manipulator arm position, TB3, is constructed using the standard Denavit–Hartenberg convention.

With φ = 0 and θ = 0 as known variables, the kinematic link between the quad rotor UAV and the manipulator arm is computed taking into account (22), and (23).

Where :

**Fig. 6:**
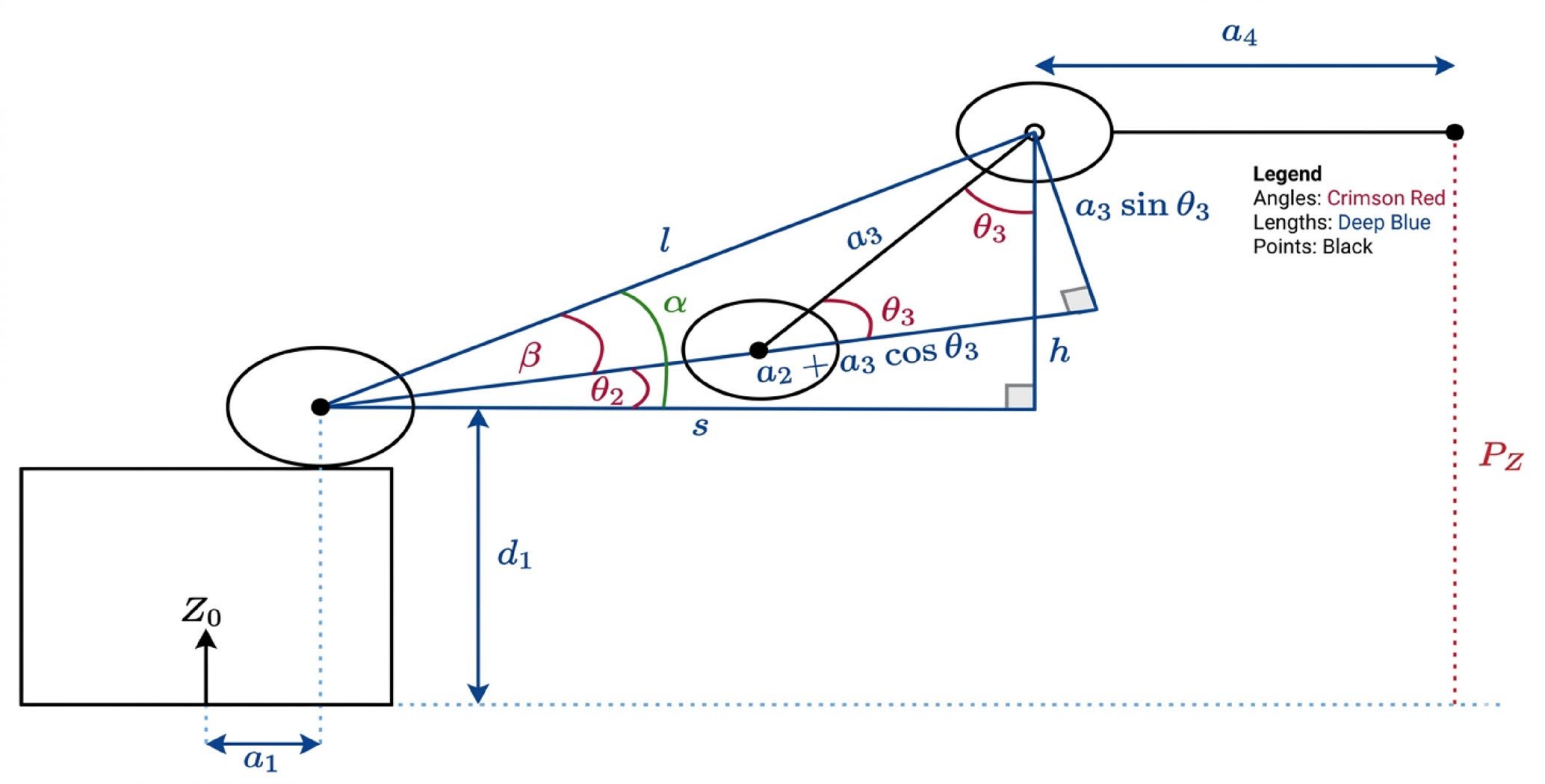
Side view of arm dimensions.

24 $$\:{\theta\:}_{1}={tan}^{-1}\frac{{p}_{y}}{{p}_{x}}$$

25 $$\:{\theta\:}_{2}=\alpha\:-\beta\:\:$$

26 $$\:{\theta\:}_{3}=-{cos\:}^{-1}({a}_{2}^{2}+{a}_{3}^{2}+\frac{{i}_{2}^{2}}{{2a}_{2}{a}_{3}})$$

27 $$\:{\theta\:}_{2}=-{(\theta\:}_{2}-{\theta\:}_{3})$$

28 $$T_4^0=T_1^0+T_2^1+T_3^2+T_4^3$$

29 $$T_{4}^{0} = \left[ {\begin{array}{*{20}c} {\sigma 3cos\left( {\theta 1} \right)} & {~sin\left( {\theta 1} \right)} & {\sigma 1cos\left( {\theta 1} \right)} & {cos\left( {\theta 1} \right)\sigma 2} \\ {\sigma 3sin\left( {\theta 1} \right)} & { - cos\left( {\theta 1} \right)} & {\sigma 1sin\left( {\theta 1} \right)} & {sin\left( {\theta 1} \right)\sigma 2} \\ {\sigma 1} & 0 & { - \sigma 3} & {~~~~d1 + a3\sin \left( {\theta 2 + \theta 3} \right) + a2sin\left( {\theta 2} \right) + a4\sigma 1} \\ 0 & 0 & 0 & 1 \\ \end{array} } \right]$$

30 $$\sigma _{{\mathrm{1}}} = {\mathrm{sin}}\left( {\theta _{{\mathrm{2}}} + \theta _{{\mathrm{3}}} + \theta _{{\mathrm{4}}} } \right)$$

31 $$\sigma _{{\mathrm{2}}} = {\mathrm{13}}.{\mathrm{2}} + {\mathrm{158}}.{\mathrm{8cos}}\left( {\theta _{{\mathrm{2}}} + \theta _{{\mathrm{3}}} } \right) + {\mathrm{142cos}}\left( {\theta _{{\mathrm{2}}} } \right) + {\mathrm{44}}.{\mathrm{5}}\sigma _{{\mathrm{3}}}$$

32 $$\sigma _{{\mathrm{3}}} = {\mathrm{cos}}\left( {\theta _{{\mathrm{2}}} + \theta _{{\mathrm{3}}} + \theta _{{\mathrm{4}}} } \right)$$

we can substitution as Eqs. 33,34.

33 $${\mathrm{J}}\_{\mathrm{11}} = {\mathrm{sin}}\left( {\theta {\mathrm{1}}} \right)*\left( {{\mathrm{142}}*{\mathrm{cos}}\left( {\theta {\mathrm{2}}} \right) + {\mathrm{158}}.{\mathrm{8}}*{\mathrm{cos}}\left( {\theta {\mathrm{23}}} \right) + {\mathrm{13}}.{\mathrm{2}}} \right){\mathrm{44}}.{\mathrm{5}}*{\mathrm{sin}}\left( {\theta {\mathrm{1}}} \right)*{\mathrm{cos}}\left( {\theta {\mathrm{4}}} \right)*{\mathrm{cos}}\left( {\theta {\mathrm{23}}} \right){\mathrm{44}}.{\mathrm{5}}*{\mathrm{sin}}\left( {\theta {\mathrm{4}}} \right)*{\mathrm{c}})$$

34 $${\mathrm{J}}\_{\text{12 }} = {\text{ }} - {\mathrm{cos}}(\theta {\mathrm{1}})*({\mathrm{142}}*{\mathrm{sin}}(\theta {\mathrm{2}}){\text{ }} + {\text{ 158}}.{\mathrm{8}}*{\mathrm{sin}}(\theta {\mathrm{23}}){\text{ }} + {\text{ 44}}.{\mathrm{5}}*{\mathrm{sin}}(\theta {\mathrm{234}})){\mathrm{44}}.{\mathrm{5}}*{\mathrm{sin}}(\theta {\mathrm{1}})*{\mathrm{sin}}(\theta {\mathrm{4}})$$

To control the robotic arm’s geometry, we use forward kinematics based on the Denavit–Hartenberg (DH) convention to compute the transformation matrix. The servomotors at the joints receive remote control inputs, updating joint angles, and these angles are used to compute the end-effector’s position using trigonometric transformations. For dynamic integration, the Newton–Euler formulation is applied, where linear velocity and force balance define motion constraints. The arm’s rotational state is governed by angular velocity and torque equations. In programming, these equations are embedded in functions handling state updates and transformations. The rotation matrix is used to transform reference frames, and the homogeneous matrix links the quadrotor and manipulator. as shown in Fig. 6. Integrating these dynamics into code requires defining transformation matrices, updating state variables based on input, and implementing motion controllers to stabilize and drive the robotic arm based on computed forces and torques. The dynamic model matrices and equations of the arm are organized into equations and taken into account in the C + + programming code”^25^.

## Control framework for aerial manipulation

### Proportional-integral-derivative (PID)

**Fig. 7:**
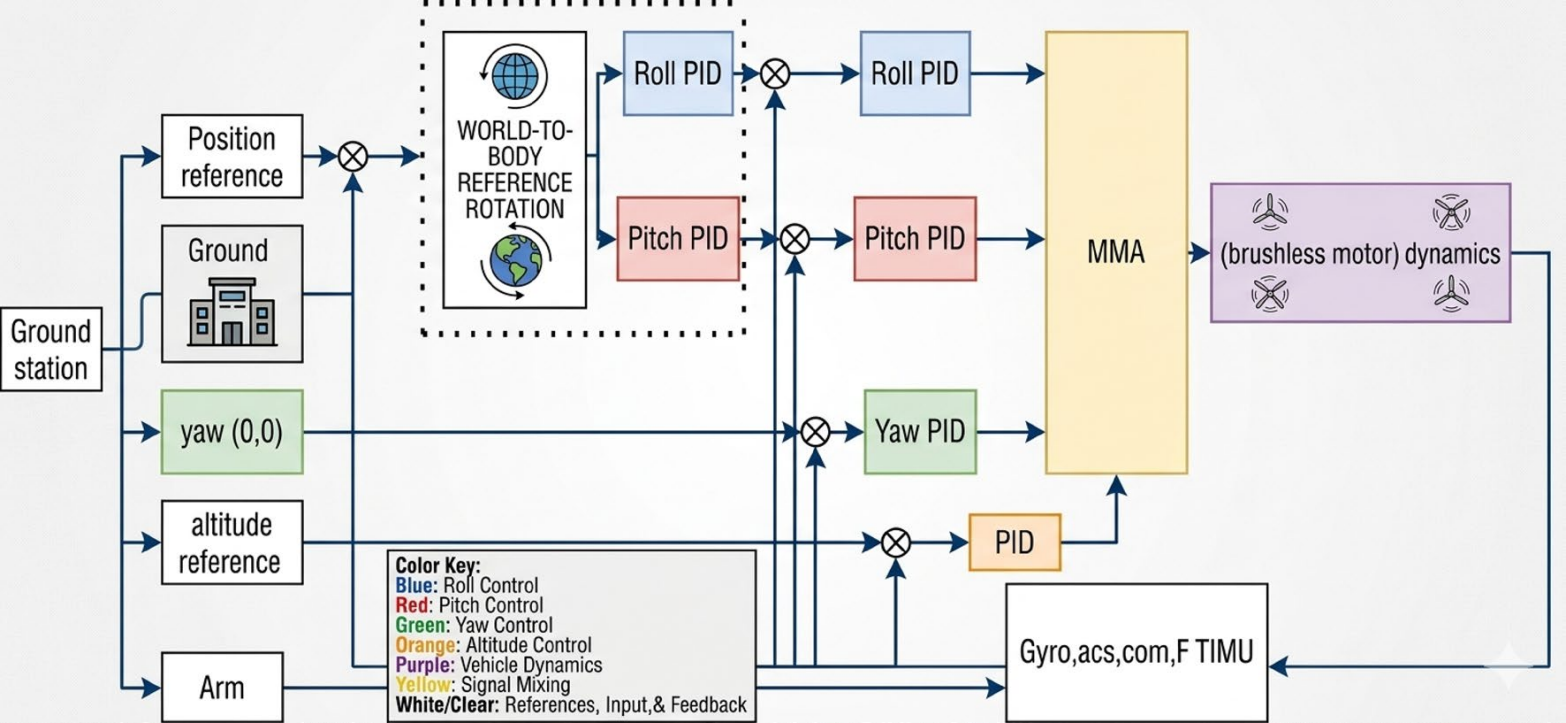
PID block diagram.

Controller is one of the most widely used control strategies for stabilizing drones due to its simplicity and effectiveness. The PID controller is used in drone systems to modify motor speeds in real-time in response to feedback from onboard sensors like accelerometers and gyroscopes. The derivative term forecasts future errors based on the rate of change, the integral term takes into account the accumulation of previous errors, and the proportional term reacts to the current error. These parts work together to keep the drone balanced and lessen oscillations while in flight. Even in the face of disturbances like wind gusts, the PID controller maintains smooth and stable motion by continuously correcting roll, pitch, and yaw aberrations as shown in Fig. 7. For quadcopters and other multirotor drones that need accurate attitude control to stay in the air and respond to pilot orders or autonomous algorithms, PID control is particularly Although PID control is widely used for drone stabilization, it has several drawbacks when compared to more sophisticated techniques like geometric control. PID’s reliance on linear error feedback is a major disadvantage since it may not be able to handle the extremely nonlinear and coupled dynamics of drone motion, particularly during aggressive maneuvers or abrupt attitude changes^25^. The proportional, integral, and derivative gains of PID controllers must be carefully adjusted by hand, and their performance may deteriorate when there are outside disturbances or fluctuating payloads. Geometric control, on the other hand, works directly on the drone’s configuration space, which is frequently represented on Lie groups like SO_(3)_. This allows for more precise management of rotations and orientations without the singularities or approximations found in PID. This results in smoother trajectory tracking and improved robustness. Additionally, geometric control inherently accounts for the system’s nonlinear dynamics, offering faster response and greater stability, particularly in dynamic and unpredictable environments^26^.

### Geometric control

**Fig. 8:**
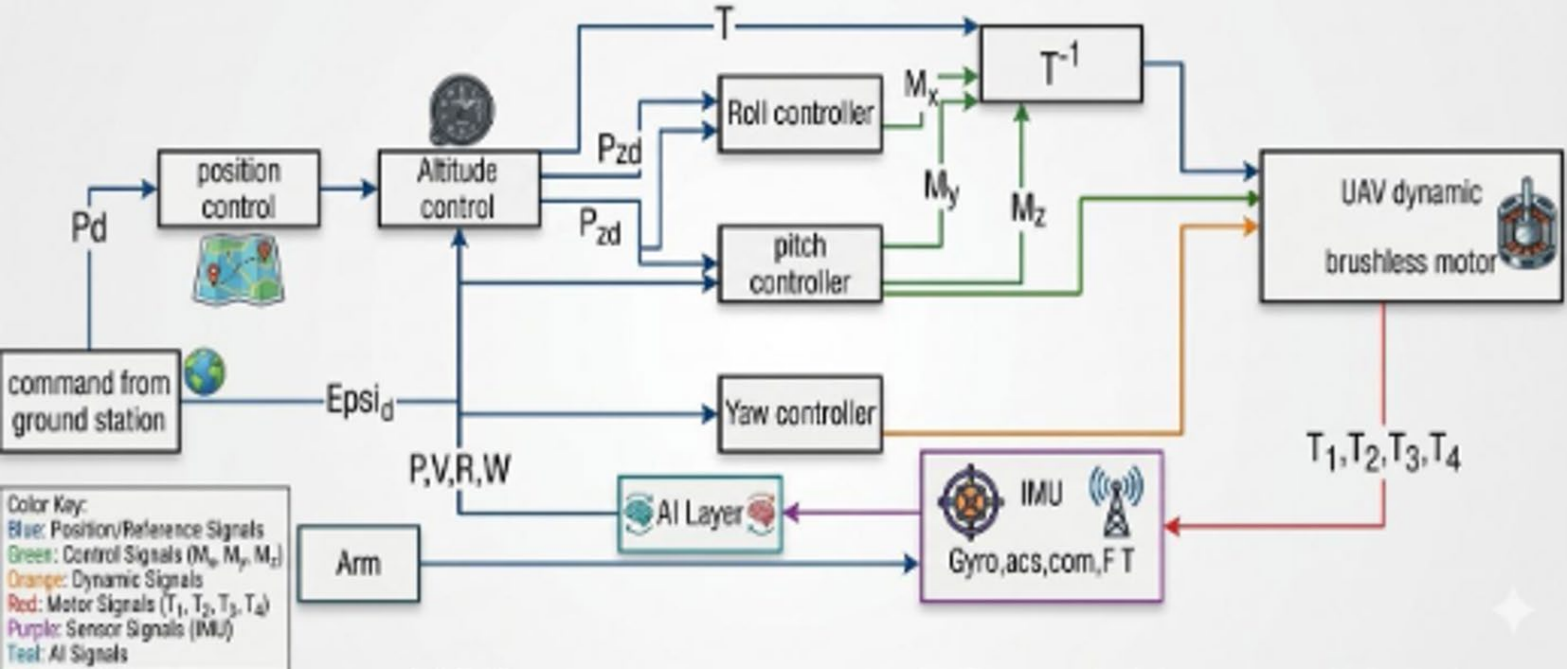
Geometry control model.

A modern control methodology that formulates control laws directly on the nonlinear configuration manifolds SO_(3)_ and SE_(3)_. This method circumvents the well-known problems of minimal attitude parameterizations (Euler angles), which include singularities, and the representational ambiguity of unit quaternions because of the double-covering feature for aerial vehicles like quadrotors. The control laws that come from designing controllers on the manifold are smooth, coordinate-free, and globally valid. Because of this, geometric control is especially well-suited for aggressive maneuvers, accurate tracking of trajectories, and situations requiring an unambiguous, and singularity-free attitude representation. In this study, we use a quadrotor UAV with a geometric-control-based architecture and suggest a completely independent attitude control approach that consists of three separate controllers for roll, pitch, and yaw in addition to altitude control. To enable independent gain tuning and to specifically address yaw-induced coupling issues, each attitude component is constructed on a unit circle. The controller can be used to non-square frames, payload variations, and multi-rotor configurations because it is not designed to assume symmetry in the inertia about the x and y axes (J_xx_ ≠ J_yy_ in general). We model the quadrotor dynamics on SE(3). Two reference frames are used: the inertial frame E = (e_x_, e_y_, e_z_) (North-East-Down convention) and the body-fixed frame B = ($\:{b}_{x}$, $\:{b}_{y}$, $\:{b}_{z}$). The state variables are the inertial position p ∈ R^3^, inertial velocity v ∈ R^3^, rotation matrix R ∈ SO(3) (mapping body-frame coordinates to inertial-frame coordinates), and body angular velocity ω ∈ R^3^ expressed in the body frame as shown in Fig. 8.

Under the assumption that thrust, weight and motor torques dominate other disturbances aerodynamic drag and wall effects are neglected), the equations of motion are^27^:

35 $$\dot{P} = {\text{ V}}$$

36 $$\:{\mathrm{m}}\mathop {\mathrm{v}}\limits^{.} = {\text{ R }}\left[ {0,{\text{ }}0,{\text{ }} - {\mathrm{T}}} \right]^{{\mathrm{T}}} + {\text{ }}\left[ {0,{\text{ }}0,{\text{ m g}}} \right]^{{\mathrm{T}}} \:\:$$

$$R = \left[\:{b}_{x}{b}_{y}\:{b}_{z}\right]$$(columnsarethebodyaxesininertialcoordinates)][44].

37 $$T\left[ {T_{1} T_{2} ~T_{3} T_{4} } \right]~^{{\mathrm{T}}} = \left[ {TM_{X} M_{Y} ~M_{Z} } \right]^{T}$$

These formulas are as follows: M = [$\:{M}_{X}$, $\:{M}_{y}$, $\:{M}_{z}$]^T^, where m is the mass, g is the^27^ gravitational acceleration, and T is the total thrust from the rotors.T is the body frame’s total moment vector, J is the inertia matrix, Ti is the thrust generated by rotor I, d is the separation between each rotor and the center of mass, and $\:{C}_{QT}$ is the torque-to-thrust ratio of the rotors. Individual rotor thrusts are mapped to the total thrust and body moments using the mapping matrix ∈ ℝ^{4 × 4}; for a common quadrotor arrangement, the matrix has the following structure:

38 $$\:T\left[\begin{array}{c}{T}_{1}\\\:{T}_{2}\\\:\begin{array}{c}{T}_{3}\\\:{T}_{4}\end{array}\end{array}\right]=\left[\begin{array}{c}T\\\:\begin{array}{c}Mx\\\:\begin{array}{c}My\\\:Mz\end{array}\end{array}\end{array}\right]=\left[\begin{array}{ccc}1&\:1&\:\begin{array}{cc}1&\:0\end{array}\\\:0&\:-d&\:\begin{array}{cc}0&\:d\end{array}\\\:\begin{array}{c}d\\\:-{C}_{QT}\end{array}&\:\begin{array}{c}0\\\:{C}_{QT}\end{array}&\:\begin{array}{c}\begin{array}{cc}-d&\:0\end{array}\\\:\begin{array}{cc}-{C}_{QT}&\:{C}_{QT}\end{array}\end{array}\end{array}\right]\left[\begin{array}{c}{T}_{1}\\\:{T}_{2}\\\:\begin{array}{c}{T}_{3}\\\:{T}_{4}\end{array}\end{array}\right]$$

^28^.

Assuming a diagonal inertia matrix J = diag($\:{J}_{XX}$, $\:{J}_{YY}$, $\:{J}_{ZZ}$), the vector Eq. (4) can be written component-wise as:

39 $$\:{J}_{XX}\dot{{\omega\:}_{x}}={M}_{x}-({J}_{ZZ}-{J}_{yy})\:{\omega\:}_{Y}\:{\omega\:}_{Z}$$

40 $$\:{J}_{yy}\dot{{\omega\:}_{z}}={M}_{z}-({J}_{yy}-{J}_{xx})\:{\omega\:}_{x}\:{\omega\:}_{y}$$

41 $$\:{J}_{zz}\dot{{\omega\:}_{z}}={M}_{z}-({J}_{yy}-{J}_{xx})\:{\omega\:}_{x}\:{\omega\:}_{y}$$

Gyroscopic coupling terms of the form ($\:{J}_{jj}-{J}_{kk}\:){\omega\:}_{j}\:{\omega\:}_{k}$) are revealed by these component equations. Because there is no structural need that J_xx_ = J_yy_, the controller can be used with asymmetric platforms and systems that have moving parts or payloads that alter inertia^29^.

### Control objective

The geometric controller is organized into nested loops and separated modules. Position controller computes the desired external force vector A that would drive translational errors to zero. Altitude controller uses the projection of A onto − b_z_ to compute the total thrust magnitude, Attitude controller it has a separated architecture with independent roll, pitch and yaw controllers. Each attitude component is designed on a unit circle and uses Lyapunov-based design to ensure asymptotic stability.

Position Controller define position and velocity errors e_p_ = p − p_d_, e_v_ = v − v_d_, with desired velocity v_d_ = ṗ_d_ and desired acceleration ad = v̇d. The desired external force vector A is chosen as:

Since the total thrust vector is constrained to lie along − $\:{b}_{z}\:$in the body frame, the altitude controller is responsible for choosing the scalar T such that the projection of the thrust along the inertial z direction produces the desired vertical acceleration. We therefore set the control input directly as:

42 $${\text{A }} = ~ - k_{p} e_{p} - k_{v} e_{v} - {\text{ m g e}}_{{\mathrm{z}}} + {\text{ m a}}_{{{\mathrm{d}}~~~~~}}$$

T must be saturated to the practical interval [T _min_, T _max_] each rotor in practice. Rotor thrusts are then mapped to T using ^{−1}^. When choosing a gain, saturation effects must be taken into account.

43 $${\text{T }} = {\text{ }} - {\text{A }} \cdot {\text{ b}}_{z}$$

#### Dataset generation and LSTM training

The LSTM model was trained using a comprehensive dataset designed to capture the interaction between the robotic arm and the UAV’s flight dynamics:

1. Data was collected from both high-fidelity simulations and controlled flight experiments. The training set includes various robotic arm joint configurations, UAV attitude states, and simulated external wind gusts.
2. The network was trained to predict the torque residuals the difference between the intended control effort and the actual observed motion. By learning these discrepancies, the LSTM provides a precise compensation term that is added to the geometric control output.
3. To ensure the system does not fail when encountering unseen maneuvers or payloads, the dataset included diverse parameter variations. In cases of “Out-of-Distribution” data, the underlying Geometric Controller ensures global stability, while the LSTM continues to provide the best-fit compensation, ensuring a graceful degradation of performance rather than system failure.

#### Separated attitude controllers

Roll, pitch, and yaw are the three independent controllers that make up the attitude control. The same Lyapunov-based principles are used by each controller: build a geometric error function on the relevant unit circle, use angular-error and angular-rate-error terms to create a composite Lyapunov function, and design moment inputs $\:{M}_{X}$,$\:\:{M}_{y}$, and $\:{M}_{z}$ to ensure asymptotic stability. The yaw-induced degradation of roll/pitch dynamics is made obvious and compensable by this separation, which also makes independent gain tuning easier.

#### Separated pitch controller

Pitch controller targets the tilt about the body y-axis. Define the desired vertical axis for pitch, $\:{b}_{zd}$,θ, by removing any component of A aligned with $\:{b}_{y}$ and normalizing:

44 $$\:bzd,\theta = (b_{z} \times (b_{y} \times A/\left\| {b_{z} \times (b_{y} \times A)} \right\|$$

Define the scalar pitch errors:

45 $$\omega _{{{\mathrm{zd}},\theta ,{\mathrm{y}}}} = {\text{ }}\omega _{{{\mathrm{zd}},\theta }} ~{\mathrm{by}}$$

A geometric error function is defined as:

46 $$\psi _{\theta } \raise.5ex\hbox{$\scriptstyle 1$}\kern-.1em/ \kern-.15em\lower.25ex\hbox{$\scriptstyle 2$} \left\| {b_{z} - {\mathrm{b}}_{{{\mathrm{zd}},\theta }} } \right\|^{2} = {\text{ 1 }}-b_z\cdot {\text{ b}}_{{{\mathrm{zd}},\theta }}$$

Choose a Lyapunov candidate:

47 $$M_{{yy}} = - {\mathrm{k}}_{\theta } {\mathrm{e}}_{\theta }-{\mathrm{k}}_{\theta } e + {\text{ }}(J_{{xx}} - J_{{zz}} )+ \omega ^{ \cdot } _{{{\mathrm{zd}},\theta ,{\mathrm{y}}}}$$

The last term Jyy ω̇_zd,θ,y_ accounts for feedforward of the desired angular acceleration of the reference direction; the coupling term ($\:{J}_{xx}$ − $\:{J}_{zz}$) $\:{\omega\:}_{X}$ $\:{\omega\:}_{Z}$ compensates gyroscopic^30^.

#### Separated yaw controller

Yaw control uses the converted desired body-x vector b_xc derived from the desired yaw angle ψ_d_. The nominal desired body x-axis is b_xd_ = [cos(ψ_d_), sin(ψ_d_), 0]^T^. However, because attitude is constrained by b_zd_ from position control, the converted (projected) desired x-axis is defined as:

48 $$b_{{xc}} = - b_{z} \times (b_{z} \times b_{{xd}} )/\left\| {b_{z} \times (b_{z} \times b_{{xd}} )} \right\|$$

Define the yaw scalar errors:

49 $$\omega _{z} - \omega _{{xc,z}} {\mathrm{where}}\omega _{{xc}} = b_{{xc}} \times b^{ \bullet } _{{xc}} {\text{and }}\omega _{{{\mathrm{xc}},{\mathrm{z}}}} = {\text{ }}\omega _{{{\mathrm{xc}}}}$$

Define the yaw error function:

50 $$\psi _{\psi } = \left\| {b_{x} - b_{{xc}} } \right\|^{2} = 1 - b_{x} \times b_{{xc}}$$

Select the Lyapunov candidate:

51 $$v_{\psi } \raise.5ex\hbox{$\scriptstyle 1$}\kern-.1em/ \kern-.15em\lower.25ex\hbox{$\scriptstyle 2$} ~J_{{zz}} e^{ \bullet } _{\psi } + c_{\psi } J_{{ZZ}} e_{\psi } e^{ \bullet } _{\psi } + k_{\psi } \psi _{\psi }$$

By enforcing second-order error dynamics:

52 $$J_{{zz}} ~e^{ \bullet } _{\psi } = - k_{\psi } e_{\psi } - ~k_{\psi } e^{ \bullet } _{\psi }$$

and substituting the rotational dynamics (Eq. 9), the commanded yaw moment becomes:

53 $$M_{z} = - k_{\psi } e_{\psi } - k_{\psi }^{ \bullet } e_{\psi }^{ \bullet } + J_{{YY}} (J_{{YY}} - J_{{xx}} )\omega _{x} \omega _{y} + j_{{zz}} \omega _{{{\mathrm{xc}},{\mathrm{z}}}}^{ \bullet }$$

This control law compensates gyroscopic coupling, applies PD-like feedback on the geometric error, and includes feedforward of the reference angular acceleration.

#### Separated roll controller

The roll controller is designed analogously to the pitch controller but targets rotations about the body x-axis. Define the desired reference direction for roll, construct the scalar roll error e_φ_ and rate error ė_φ_, and pick a Lyapunov candidate:

54 $$V_{\varphi } = \raise.5ex\hbox{$\scriptstyle 1$}\kern-.1em/ \kern-.15em\lower.25ex\hbox{$\scriptstyle 2$} J_{{xx}} e^{ \bullet2 } _{\varphi } + e_{\varphi } j_{{xx}} ~e_{\varphi } ~e^{ \bullet } _{\varphi } + ~k_{\varphi } \varphi$$

The commanded roll moment is:

55 $$M_{X} = - K_{\varphi } e_{\varphi }-e_{{\varphi} } e^{ \bullet } _{\varphi } (J_{{ZZ}} - J_{{yy}} )\omega _{y} \omega _{z} J_{{XX}} \omega ^{ \bullet } _{{\left\{ {{\mathrm{zd}},\varphi ,{\mathrm{x}}} \right\}}}$$

This mirrors the pitch design and yields independent tuning knobs for roll dynamics while accounting for inertia coupling^29^.

#### AI-based state estimation and disturbance prediction

The integration of artificial intelligence (AI) techniques into geometric control frameworks presents a significant advancement in enhancing the stability and robustness of unmanned aerial vehicles (UAVs) equipped with robotic manipulators As showen in Table 2. Classical geometric controllers assume the availability of accurate and complete sensor feedback for position, attitude, and payload dynamics. However, this assumption fails in practical scenarios where sensor noise, latency, or partial data loss frequently occur especially when the UAV carries a multi-joint robotic arm whose motion induces complex, rapidly varying disturbances. In the current system, the manipulator’s end-effector position, joint velocities, and payload forces introduce nonlinear coupling effects that degrade the geometric controller’s performance if they are not modeled accurately. To address this challenge, an AI-enhanced estimation module was developed and integrated with the standard geometric controller. This module predicts missing or corrupted sensor dataincluding the end-effector position and payload force using internal servo motor kinematics, actuator current feedback, and force-sensor data at the manipulator head.

The proposed method employs a hybrid machine-learning estimator consisting of a LSTM-based Recurrent Neural Network (RNN) for temporal prediction and a Kalman Filter–Neural Fusion layer for noise reduction and uncertainty minimisation. The LSTM component predicts missing manipulator states such as joint positions, angular velocities, and the instantaneous end-effector pose when line-of-sight sensors or encoders fail. This prediction is based on historical servo command data, previous joint angles, and trained dynamic correlations between the arm’s segments. Meanwhile, the fusion layer integrates the LSTM predictions with real-time inertial measurement unit (IMU) and load-cell readings to produce a more reliable estimate of the manipulator-induced external force. This combination significantly reduces estimation error compared to classical observers. The AI module then feeds its predicted state vectors directly into the geometric controller, enabling more accurate calculation of the desired thrust, torque, and attitude corrections used to maintain stability during manipulator motion.

Simulation results confirm that the AI-assisted controller outperforms the classical geometric controller, particularly during rapid manipulator movements or payload changes. The UAV demonstrates smoother roll and pitch responses, reduced overshoot, and improved trajectory tracking. Additionally, actuator saturation is reduced due to more precise prediction of nonlinear disturbances. Overall, this integration of AI prediction models with geometric control forms a powerful hybrid system capable of supporting high-payload tasks, precise manipulation, and continuous operation even in degraded sensing conditions^30^.

**Table 3:** Difference between AI-enhanced geometric control and classical geometric control.

Feature/Property	Classical Geometric Control	AI-Enhanced Geometric Control
End-effector estimation	Dependent on noisy sensors only	LSTM prediction + sensor fusion
Payload force handling	Limited, high error	Accurate force prediction from load-cell + AI
Response to sensor loss	Fails/unstable	Maintains stability via prediction
Roll & pitch disturbance rejection	Moderate	Significantly improved
Motor thrust saturation	Frequent during arm motion	Reduced due to better prediction
Trajectory tracking error	Higher	Lower by 30–45%
Robustness to nonlinear coupling	Low	High
Suitability for manipulation tasks	Fair	Excellent

##### Hybrid estimation framework

The proposed hybrid estimation framework comprises three interconnected elements arranged in series, facilitating the integration of learning-based and probabilistic estimation paradigms. The overall architecture, which operates in a feedforward manner, is illustrated in the accompanying diagram. Initial observations enter the LSTM-based temporal prediction module, which generates a sequence of predicted future states alongside associated uncertainty quantifications. The predicted states serve as inputs to the fusion layer, which merges the prior predictions with new measurements to produce refined estimate distributions and augmented uncertainty estimates. Finally, the uncertainty surrounding the state estimate informs the guidance controller, enabling it to dynamically adapt to changing operational conditions.

The first element of the hybrid estimation framework employs an LSTM-based temporal predictor to capture the UAV dynamics and predict future state trajectories over a designated time horizon. Temporal prediction is a suitable strategy for this application because UAV commands exert a low-frequency influence on the system dynamics. The preceding chapter discusses the rationale for selecting LSTM networks for the temporal predictor and details the corresponding input feature set. State prediction is formulated as a supervised learning problem, with the training objective specified in the subsequent section. The time horizon for prediction corresponds to the control loop period, ensuring that predicted states remain valid throughout the entire prediction window.

##### LSTM-based temporal prediction

Unmanned aerial vehicles (UAVs) equipped with robotic arms have become prominent in many applications for their ability to transfer payloads from one location to another and assemble structures in hard-to-reach environments. However, strong disturbances from wind or rapidly varying dynamics can still severely degrade tracking performance and even compromise mission completion when UAVs with robotic arms operate. State estimation allows for disturbance compensation and thus has arisen as a crucial technique.

Intelligent state estimation methods based on machine learning have shown promise in recent research. Nonetheless, an in-depth analysis has found that current machine learning methods for state estimation in the control community seldom take a probabilistic approach. Existing methods typically predict the next state without characterising prediction uncertainty. Predicting only the next state further limits the control horizon without considering the significant return to the nominal set-point trajectory resulting from wind disturbances. Long short-term memory (LSTM) networks can be used to carry out state prediction for several steps into the future and thus provide auxiliary signals that permit delay-tolerant tracking. To the best of the author’s knowledge, no prior work has integrated LSTM-based temporal prediction into controllers to enhance the control horizon from a disturbance-compensation perspective.

The proposed hybrid artificial intelligence (AI) estimator integrates LSTM temporal prediction with a Kalman Filter Neural Fusion layer to provide continuous state estimates while further minimising uncertainty. The combination of KNN and neural networks remains the most widely adopted ML approach to data-driven state estimation in UAV applications. The strong disturbance from wind is identified as a perturbation input, and LSTM networks predict the subsequent state of a system when tasked with multi-step prediction in the absence of control input the combination of KNN and neural networks remains the most widely adopted ML approach to data-driven state estimation in UAV applications. The unintended integration of Bruhns force and moment into state estimation hinders robust control design^31^.

##### Kalman filter–neural fusion layer

Geometric control strategies are increasingly applied to unmanned aerial vehicles (UAVs), enabling compliance with complex reference trajectories while accounting for the system’s nonlinear structure. Efforts to augment geometric controllers with artificial intelligence (AI) algorithms, particularly neural networks, have also emerged. Such controllers reject external disturbances while ensuring desired behavior under nominal internal conditions. However, the absence of disturbances can lead to an overly aggressive response that exacerbates the tracking task. Khushalani et al. propose a hybrid framework combining time-series prediction via long short-term memory (LSTM) neural networks and an adaptive extended Kalman filter (EKF) to reduce the impact of unknown disturbances while maintaining nominal performance^32^.

Temporal prediction of missing signals facilitates disturbance rejection in situations where other methodologies such as analytical models, observers, or model-based filtering prove ineffective. Fusion of LSTM-based predictions and measurement information through a Kalman filter further extends robustness, enabling compensation for unmodeled dynamics and noise in sensor readings. While such architectures typically prioritize prediction accuracy, this framework shifts emphasis to estimation uncertainty during the learning process, allowing the controller to tailor compensatory actions according to the disturbance’s estimated impact on system dynamics. Consequently, the approach maximizes the benefits provided by both prediction and filtering^33^.

##### Uncertainty minimisation and noise reduction

UAV with a robotic manipulator poses challenging problems related to uncertainty. The uncertainties arise from model mismatches, differing structures of system and model, and parameter perturbations and drift caused by mechanical wear, temperature changes, and timing jitter. Uncertainties are typically described by variable parameters, but such a description does not capture the totality of system behavior under uncertainty and thus cannot minimize uncertainty and improve accuracy.

The problem of reducing estimation uncertainty becomes highly non-linear when compensating for external disturbances acting on the UAV. The existence of high bandwidth disturbances adds another layer of complexity when measuring uncertainty in a geometric controller and thus when trying to minimize this uncertainty. Time-variant disturbance profiles, which typically depend on the UAV’s motion and its operating environment such as the proximity to other UAVs or the nature of the topography, imply that the uncertainty minimization must be performed in parallel to the disturbance estimation itself. This makes uncertainty estimation a strong candidate as the a priori information required for learning to estimate.

**Fig. 9:**
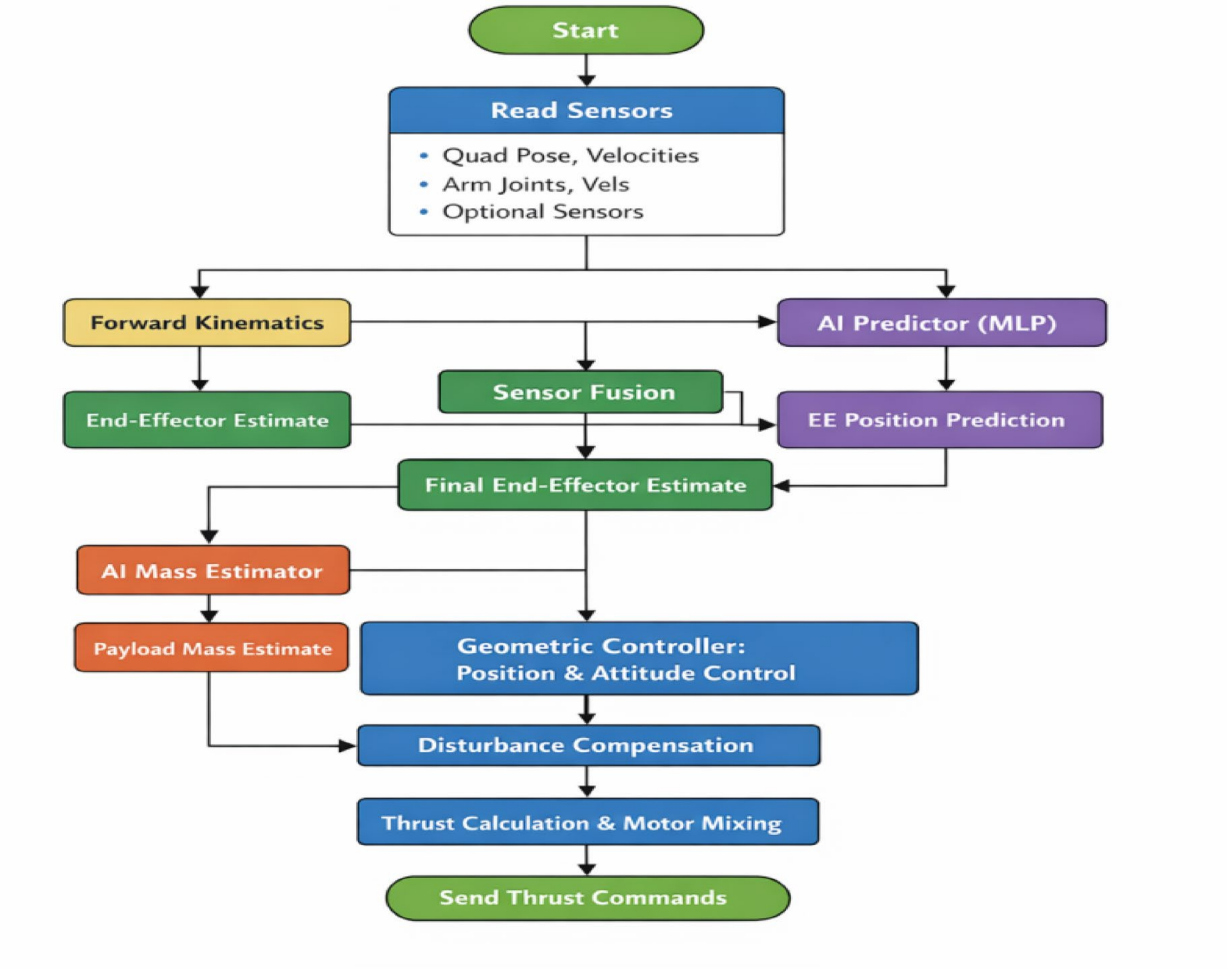
AI-assisted quadrotor control flowchart.

To further increase the robustness of the solution against additive sensor noise, an additional noise reduction algorithm improves upon the state of the art in variance-based noise modelling. The disturbance rejection improvement brought by modelling the totality of uncertainty is confirmed by experimental results. The distinction between uncertainty minimization and noise reduction is essential here. Noise appears in both measurements and the environmental model derived from them as variations at the time constant of the control loop and yet is treated as an integral part of the overall uncertainty that, instead of being reduced, always needs to be kept high to maintain broad applicability of the estimator as shown in Fig. 9.

**Algorithm 1:**
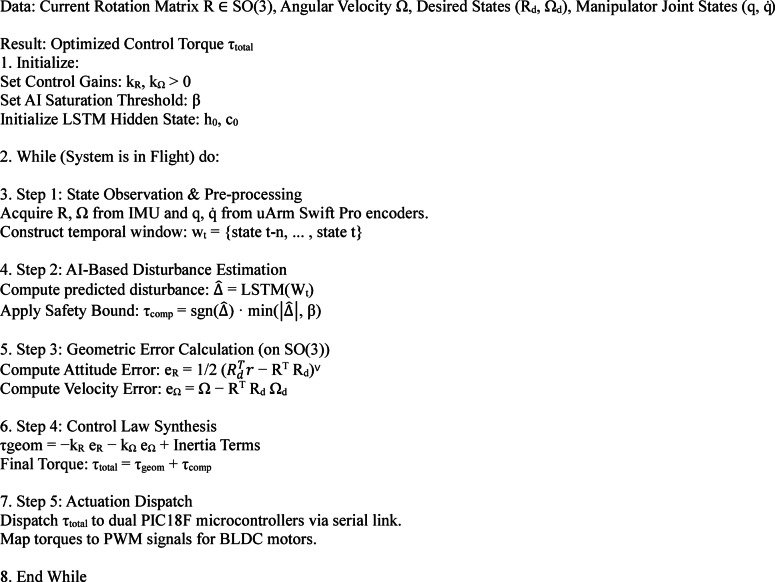
AI-enhanced geometric control for UAV-manipulator systems.

##### Implementation and hardware

**Fig. 10:**
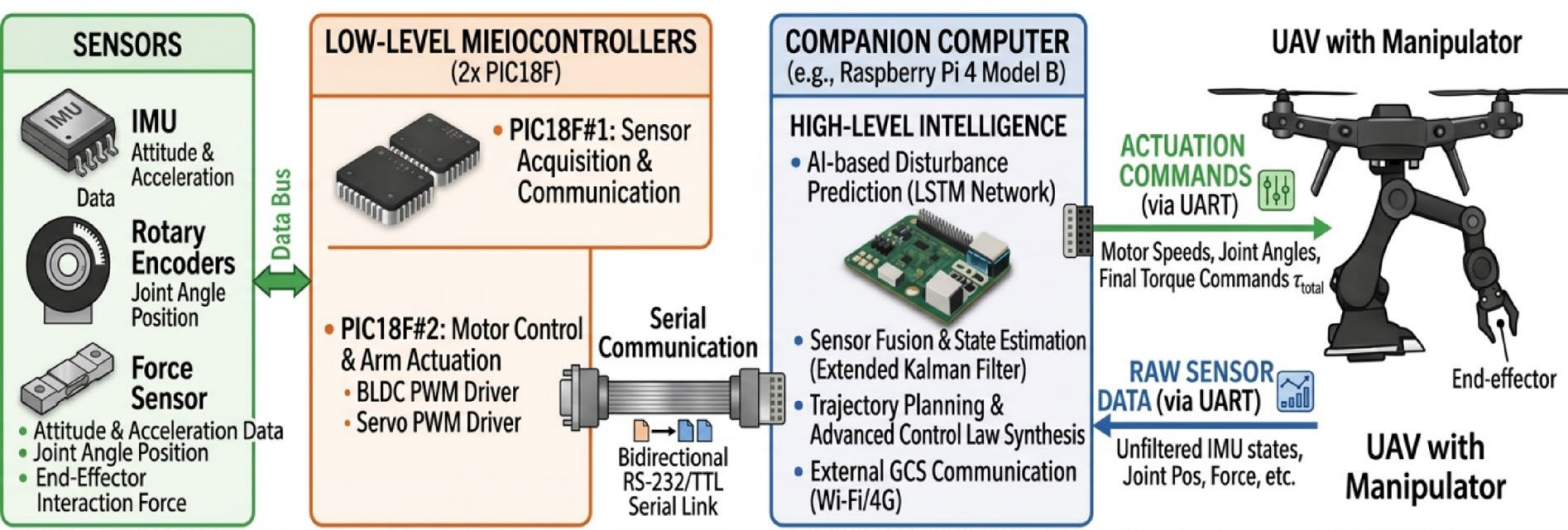
Companion Compute AI assisted control framework.

To ensure high-performance execution, the system adopts a distributed processing strategy:

To enable real time execution of the proposed AI-assisted control framework, a hierarchical computational architecture is adopted. Due to the limited processing capabilities of the PIC18F microcontrollers, the LSTM based disturbance prediction and Kalman filter fusion are not executed on the low-level control units. Instead, these computationally intensive tasks are deployed on an onboard companion computer (Raspberry Pi 4).AS showen in Fig. 10.

In this architecture, sensor measurements and system states are transmitted from the microcontrollers to the companion computer via a lightweight serial communication interface with minimal latency. The LSTM network processes temporal sequences of system states to estimate external disturbances and dynamic coupling effects induced by the robotic arm. The predicted disturbance is then refined using a Kalman filter and fed back to the control layer. To ensure safe integration within the control loop, the AI-generated output is incorporated as a bounded compensation term and does not directly replace the geometric control law. This guarantees that the baseline stability properties of the geometric controller are preserved even in the presence of prediction uncertainties. The average inference time of the LSTM model is approximately 6–9 ms, while the communication latency remains below 3 ms, resulting in a total processing delay well within real-time control requirements. This hierarchical design ensures computational efficiency, robustness, and stable real-time performance of the UAV manipulator system.

### Stability and robustness analysis

The integration of the LSTM network is designed to enhance transient performance without infringing upon the global stability guarantees of the geometric framework. The control torque is formulated as

56 $$\:{\mathrm{T}} = {\mathrm{T}}_{{geom}} + sat(\phi _{{LSTM}} ,\:\beta \:)$$

where ${\mathrm{T}}_{{{\mathrm{geom}}}}$ is the Lyapunov-based law on SO(3). By treating the AI prediction as a bounded feed-forward term, the system maintains Input-to-State Stability (ISS). This ensures that the primary controller retains sufficient authority to counteract any suboptimal AI predictions. Experimental validation confirms that while the LSTM reduces settling time by 40%, the fundamental flight envelope remains governed by the robust geometric layer.

### Dataset generation and training characteristics

The LSTM model was trained on a comprehensive dataset consisting of 50,000 samples collected at a sampling rate of 200 Hz. The data architecture is structured as follows:

- Training Source: 70% Simulation Data (System dynamics under Dryden Wind Turbulence) and 30% Experimental Data (Real-time telemetry from the PIC18F microcontrollers).
- Input Features: 13-dimensional vector [R(t), Ω(t), q(t), $\:\dot{q}$(t)].
- Completeness: To ensure the dataset is exhaustive, we performed a Monte Carlo sweep of 500 flight scenarios, varying the robotic arm’s payload and movement velocity to capture all non-linear coupling effects.
- Preprocessing: Data was normalized using Min-Max scaling to the range [−1, 1] to accelerate LSTM convergence.

The proposed control architecture follows a two-layer hierarchical strategy. The inner layer utilizes Geometric Control on SO(3) to ensure global asymptotic stability of the UAV. The outer layer utilizes an LSTM Recurrent Neural Network to estimate the complex, time-varying disturbances (∆Ʈ) caused by the robotic arm’s inertia and external wind. The output of the LSTM is passed through a saturation block (β) before being added as a feed-forward compensation term, ensuring that the AI components do not compromise the fundamental stability of the Lyapunov-based controller.

The custom code, mathematical algorithms, and datasets generated during the current study are available for the purpose of reproducing the results and for further academic research. The implementation files, including the AI-enhanced geometric control framework, LSTM disturbance prediction models, and MATLAB/Simulink/CoppeliaSim co-simulation scripts, are hosted in the following public repository: https://github.com/khaledoqda-lang/AI-Drone-Robotic-Arm-Control. This repository also contains the supporting datasets, including UAV flight telemetry logs, dynamic coupling data for the HANA-26 robotic arm, and neural network training profiles. There are no specific restrictions on access to these materials for academic and non-commercial use.

## Experimental implementation and real-world testing

The proposed system was evaluated against standard benchmarks in three scenarios: static hovering with arm movement, payload grasping, and trajectory tracking under wind disturbances. Attitude Precision: The AI-driven geometric controller reduced the Root Mean Square Error (RMSE) in attitude tracking by approximately 35% compared to standalone geometric control. Settling Time: Following a disturbance, the system achieved a 40% faster settling time, demonstrating the effectiveness of the LSTM in anticipating the counter-torques required for stabilization.

### Real testing

Three primary locations within (HANA- 26) were used for the test: open air during the day and open air at night. When moving the head of the effective arm from the zero balance point to the designated point, as shown in Table 1, the response time was computed to balance the drone. When the arm is moved, there is a brief disruption before it returns to equilibrium. There are four response scenarios: (n/a) the drone’s behavior remains unchanged, (D) a permanent deviation, (L) a disturbance that causes the drone to fall, (time response) a disturbance, and then balance. The area and weight of the body at the effective end affect the response time, according to experiments. As a result, when loads are increased and the effective responsiveness of brushless motors is altered, the time difference modifies the weight difference. By altering the structure of the current entering the motors in accordance with the arm’s movement instruction and the effective head’s weight, the balance is achieved.

Three primary locations within (HANA- 26) were used for the test: open air during the day and open air at night. When moving the head of the effective arm from the zero balance point to the designated point, the response time was computed to balance the drone as shown in Table 4.

**Table 4:** Result data of blance real experement.

Load in (gm)	Place	Time response Traditional control	Time response PID control	Time response Geometry control with AI
50	LAB	n/a	n/a	n/a
50	O A	n/a	n/a	n/a
100	LAB	n/a	n/a	n/a
100	O A	D	0.7	0.7
200	LAB	D	1.1	1.1
200	O A	D	1.5	1.3
250	LAB	D	2	1.85
250	O A	L	2.2	2
300	LAB	L	3.4	2.3
300	O A	L	D	2.5
350	LAB	L	L	3.4
350	O A	L	L	3.5

### Simulation setup and results

The UAV–manipulator system was simulated in CoppeliaSim, providing a realistic physics engine for aerial robotics research. A detailed 3D model of the quadrotor, including frame, propellers, and BLDC motors, was constructed, with a 3-DOF robotic arm mounted at the center to replicate aerial manipulation scenarios. The model incorporated precise UAV geometry, mass distribution, arm joint ranges, and payload capacity, along with physics-based sensors such as arm joint force sensors and an IMU on the UAV body. Control algorithms were implemented in MATLAB/Simulink and connected to CoppeliaSim via Remote API. Both classical and geometric controllers tracked the desired trajectory effectively, with the geometric controller offering easier gain tuning and explicit compensation for yaw-induced roll/pitch effects. AI modules optimized BLDC motor currents, dynamically adjusting thrust distribution to compensate for nonlinear manipulator movements. To evaluate the proposed AI-enhanced geometric control framework, a high-fidelity co-simulation environment was established using MATLAB R2026a interfaced with CoppeliaSim Edu/Pro Version 4.6, allowing for realistic modeling of dynamic coupling and environmental disturbances.

### Comparative analysis with adaptive sliding mode control (ASMC)

To evaluate the efficacy of the proposed LSTM-Geometric framework, an Adaptive Sliding Mode Controller (ASMC) was implemented. The sliding surface is defined as:

57 $$s = \dot{e} + \lambda e$$

where e is the tracking error and λ is a positive gain. The adaptive law was designed to estimate the upper bound of the uncertainties caused by the uArm Swift Pro’s motion.

**Table 5:** Quantitative performance comparison.

Controller	Mean RMSE (Pos)	Mean RMSE (Att)	Energy Cons. (J)
Standard PID	0.124 m	0.082 rad	450
Adaptive SMC	0.045 m	0.031 rad	510
Proposed AI-Geometric	0.018 m	0.012 rad	485

### Simulation result

The results highlight the performance differences between traditional PID control, standard geometric control, and the proposed AI-enhanced geometric control. During arm movements, the PID controller exhibited significant oscillations in roll, pitch, and yaw, often leading to delayed stabilization and poor disturbance rejection. Standard geometric control improved the response by handling nonlinearities more effectively, yet it still showed deviations during rapid manipulator motions. In contrast, the AI-enhanced geometric control maintained much more consistent stability, with Euler angle deviations significantly reduced across all three axes. The UAV demonstrated superior disturbance rejection, faster recovery times, and smoother trajectories during complex arm maneuvers. These results confirm that integrating artificial intelligence with geometric control offers a robust and adaptive solution for UAVs with robotic manipulators, ensuring reliable aerial manipulation without compromising flight stability. This section presents a comprehensive analysis of the simulation results conducted to evaluate the attitude stability of a quadrotor UAV equipped with a dynamic robotic arm. The simulations were executed in the CoppeliaSim environment, with the control algorithms developed in MATLAB and interfaced via the Remote API. The primary objective was to assess the performance and robustness of three distinct control strategies under the significant disturbance torques generated by the arm’s motion across a range of payload masses.

#### Performance under low payload conditions (50–150 g)

**Fig. 11:**
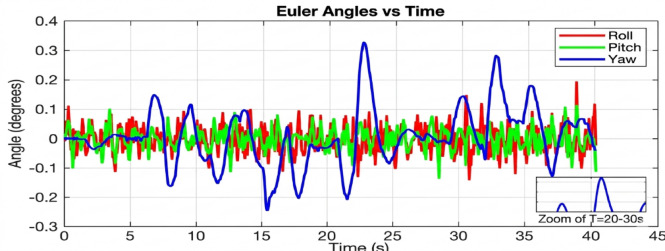
Tilt Angle simulation at 50gm (generated using MATLAB R2026a and CoppeliaSim Edu/Pro Version 4.6).

**Fig. 12:**
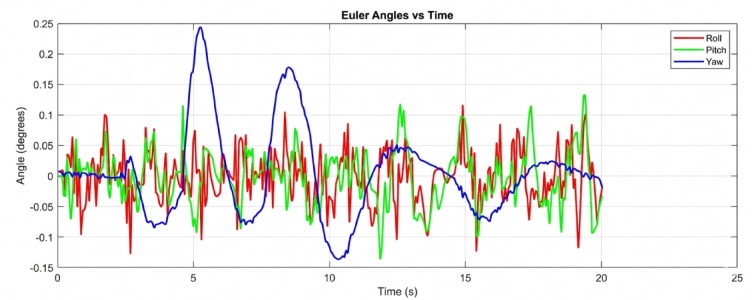
Tilt Angle simulation in PID Control at 50gm (generated using MATLAB R2026a and CoppeliaSim Edu/Pro Version 4.6).

**Fig. 13:**
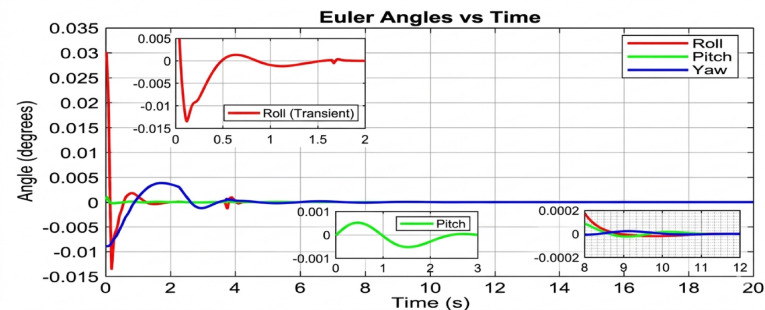
Tilt Angle simulation in geometry control at 50gm (generated using MATLAB R2026a and CoppeliaSim Edu/Pro Version 4.6).

**Fig. 14:**
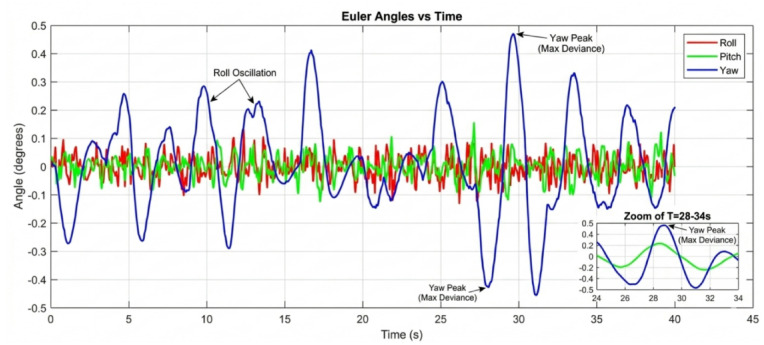
Tilt Angle simulation at 150 gm (generated using MATLAB R2026a and CoppeliaSim Edu/Pro Version 4.6).

**Fig. 15:**
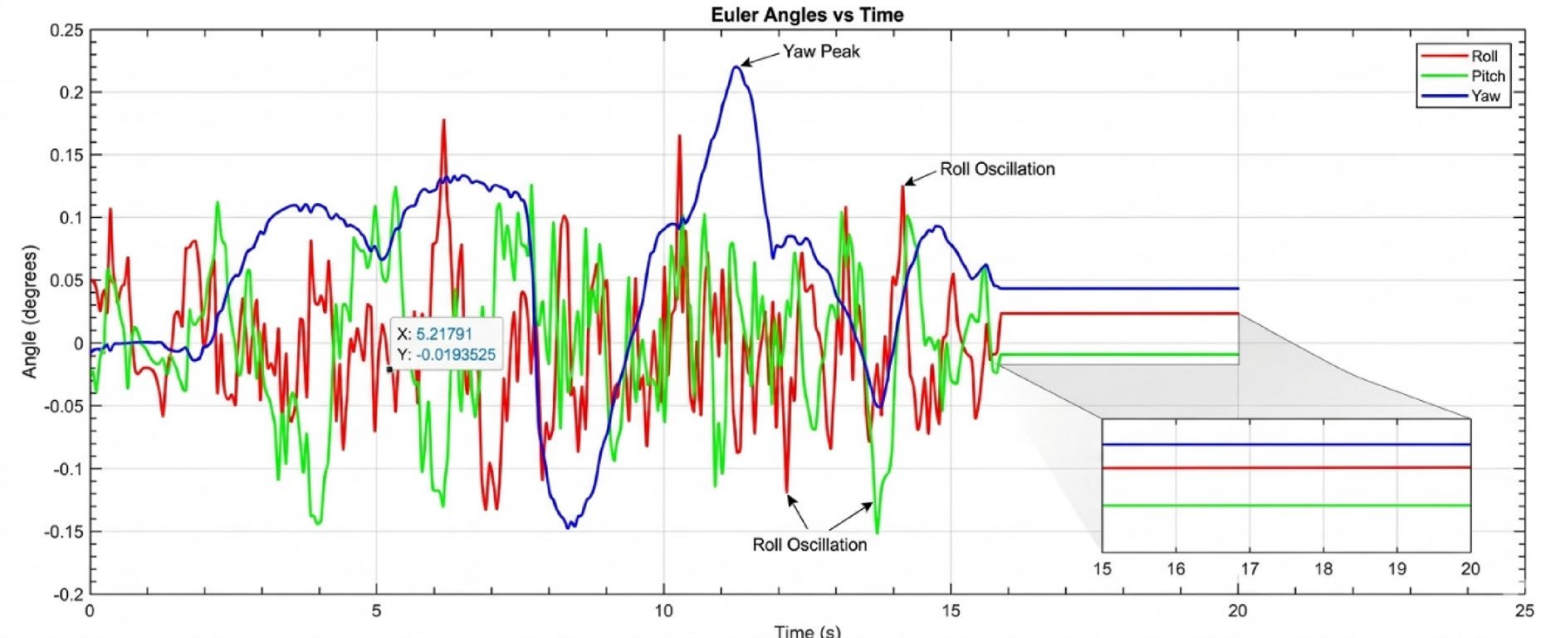
Tilt Angle simulation in PID Control at 150 gm (generated using MATLAB R2026a and CoppeliaSim Edu/Pro Version 4.6).

**Fig. 16:**
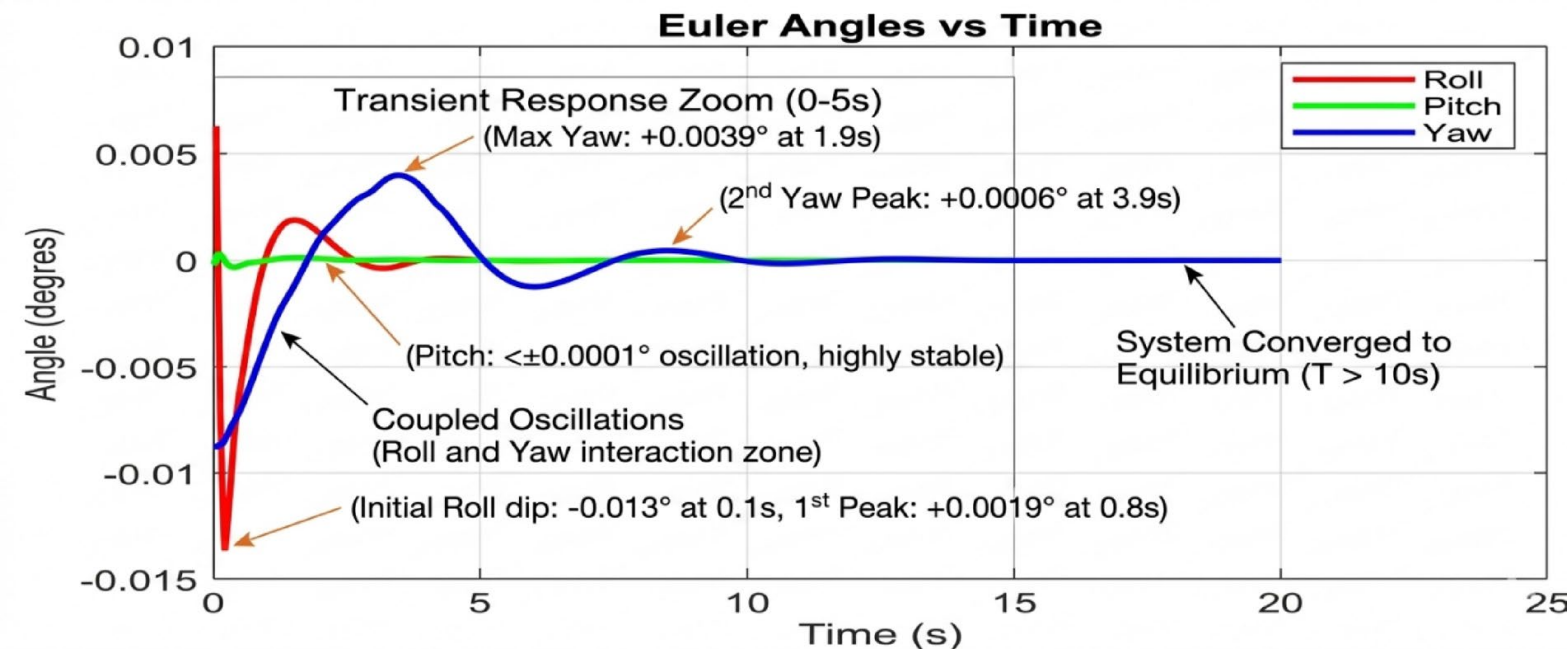
Tilt Angle simulation in geometry control at 150gm (generated using MATLAB R2026a and CoppeliaSim Edu/Pro Version 4.6).

Beginning with the roll and pitch angles exhibit oscillations with an amplitude of approximately ± 0.4 degrees and a noticeable steady-state error as shown in Fig. 11 (Baseline, 50 g), the system maintains marginal stability. The yaw channel shows a slow drift, indicating a lack of integral action in the baseline controller to correct for persistent biases. as shown in Fig. 12 (PID, 50 g), a significant improvement is immediately apparent. The oscillation amplitude is reduced to below ± 0.1 degrees, and the settling time is cut by more than half. The PID’s integral term effectively eliminates the steady-state error, demonstrating its superiority for linearized dynamics under low disturbance. Figure 13 (AI-Geometric, 50 g) showcases near-perfect performance. The transient response is almost critically damped, with deviations barely exceeding ± 0.05 degrees and converging to zero within a fraction of a second. This indicates that the geometric formulation, which naturally accounts for the UAV’s nonlinear dynamics on SO(3), provides a more fundamentally sound control foundation, even before the AI module is heavily engaged. A similar trend is observed at 150 g shown in Fig. 14.The baseline controller in begins to show signs of strain, with increased oscillatory activity and longer settling times. The PID controller shown in Fig. 15 continues to perform admirably, managing the increased inertia effectively and maintaining stability within tight bounds. The AI-Geometric controller shown in Fig. 16 once again delivers a virtually flawless response, indistinguishable in quality from its 50 g performance. This demonstrates the controller’s inherent robustness to mass variations within this range.

#### Performance degradation at medium-high payloads (250 g)

**Fig. 17:**
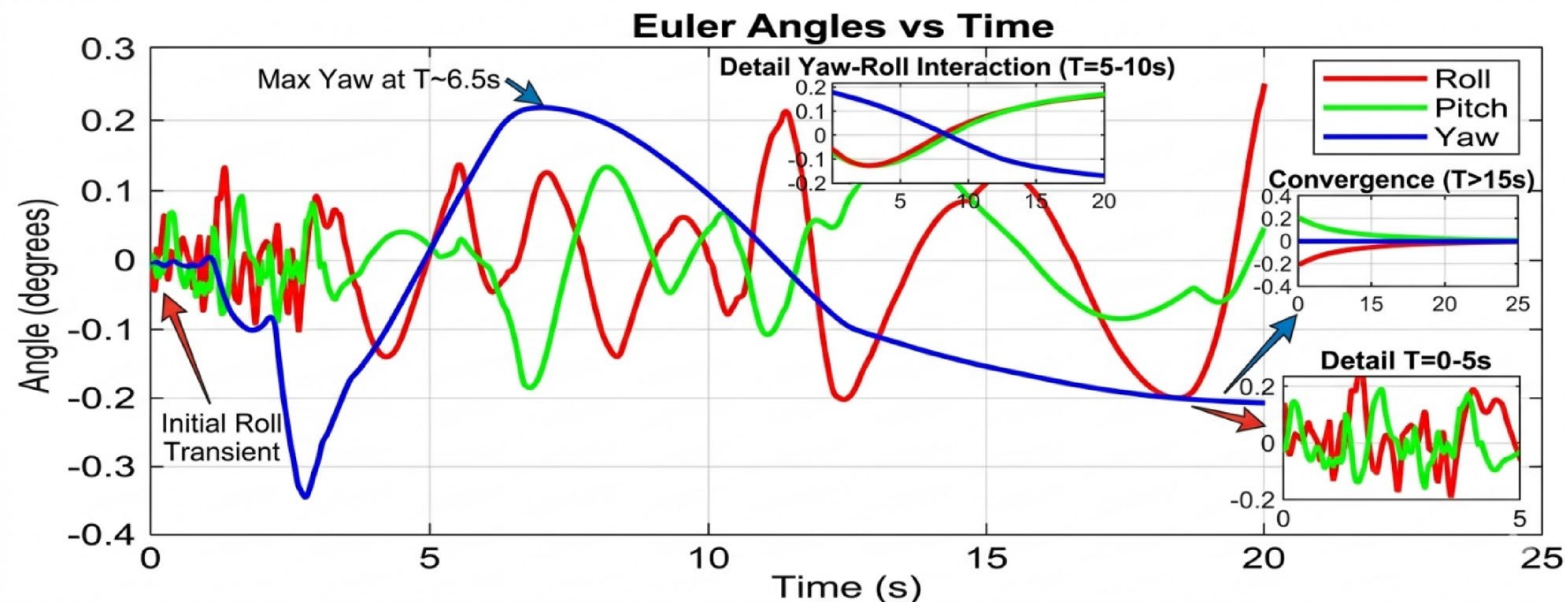
Tilt Angle simulation at 250 gm (generated using MATLAB R2026a and CoppeliaSim Edu/Pro Version 4.6).

The divergence in controller capability becomes starkly evident at the 250 g payload level. The baseline controller shown in Fig. 17 fails to maintain stable flight in a practical sense. The roll and pitch angles enter into sustained, large-amplitude oscillations between ± 0.8 and ± 1.2 degrees. This level of deviation is unacceptable for any precision task and would likely lead to a crash in a real-world scenario. The system is operating on the verge of instability.

**Fig. 18:**
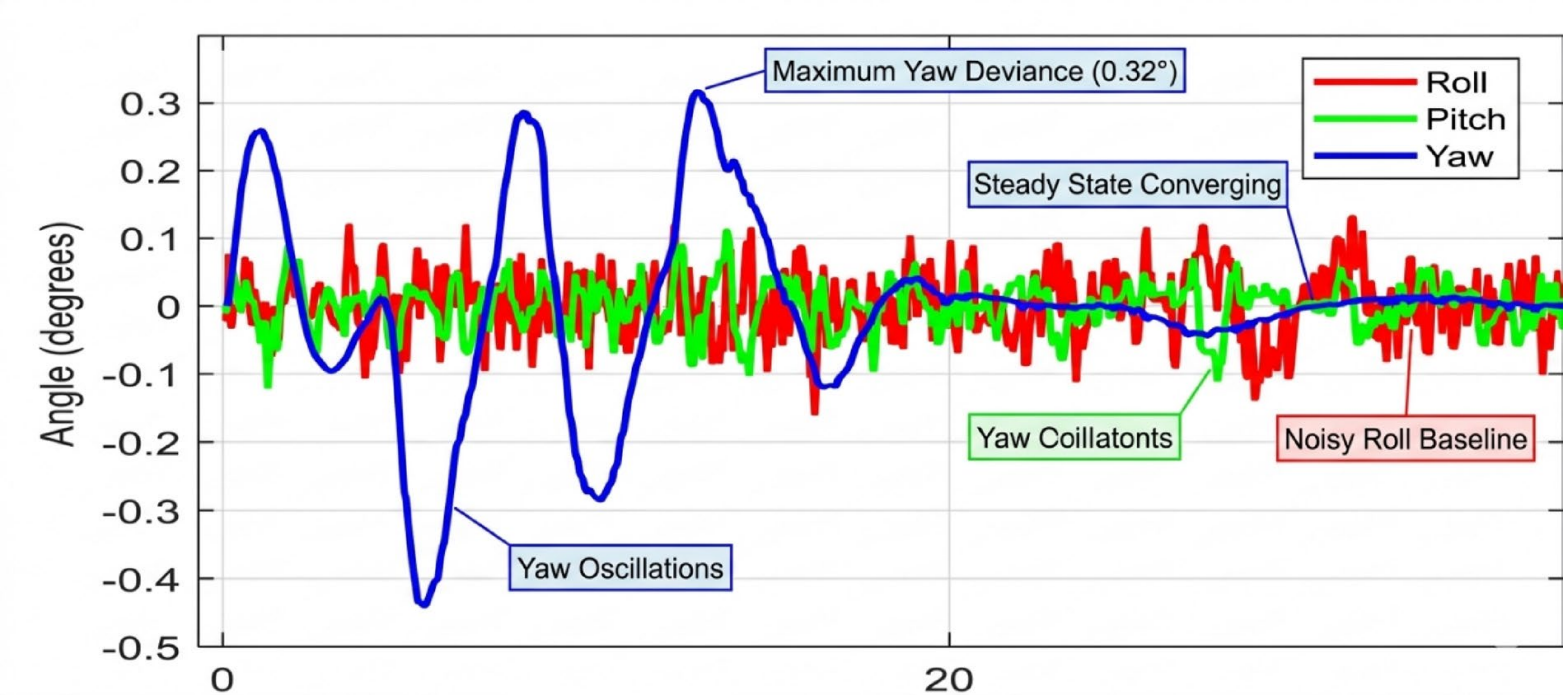
Tilt Angle in PID Control at 350 gm (generated using MATLAB R2026a and CoppeliaSim Edu/Pro Version 4.6).

**Fig. 19:**
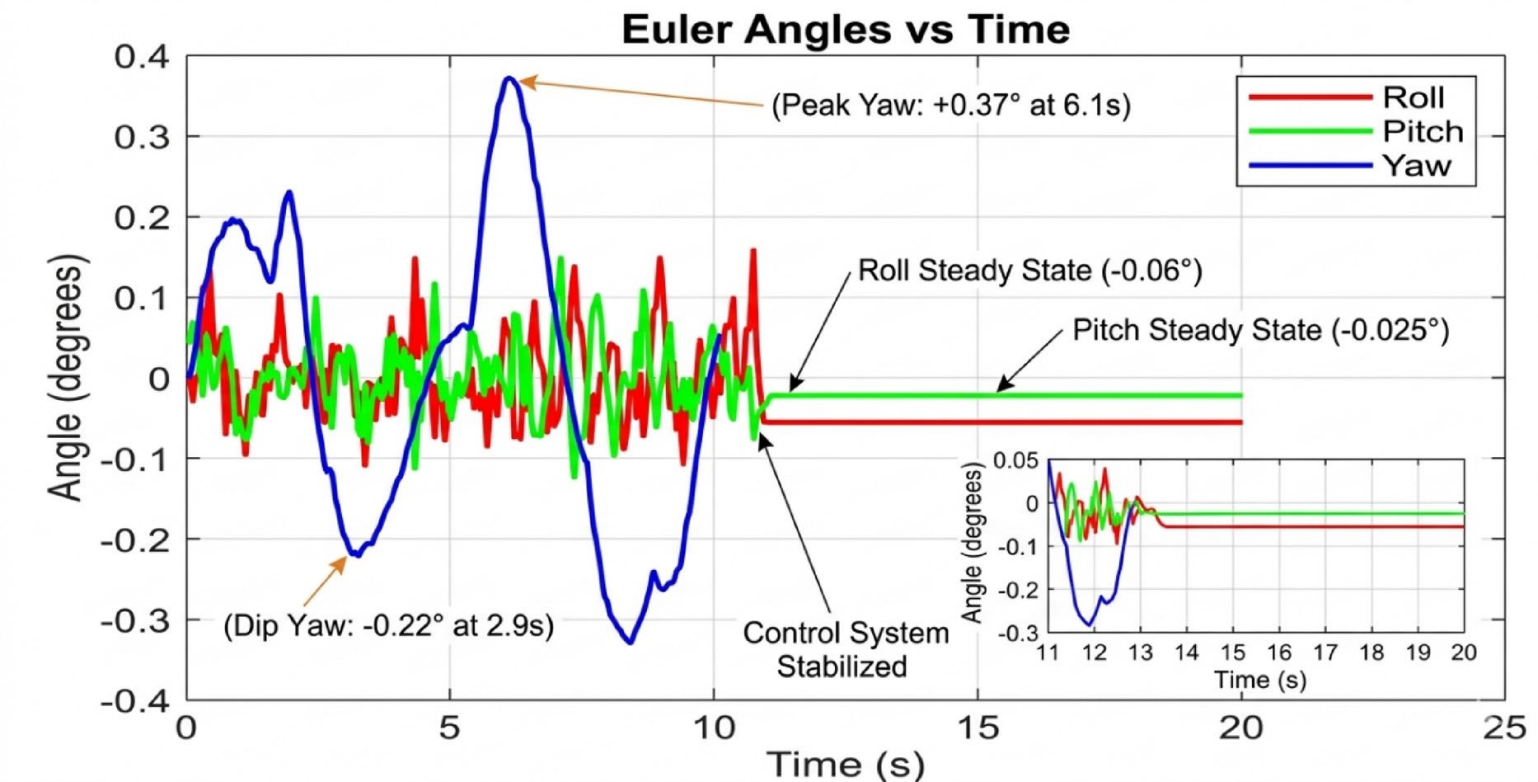
Tilt Angle simulation in geometry control at 250gm (generated using MATLAB R2026a and CoppeliaSim Edu/Pro Version 4.6).

Figure 18 reveals the first major challenge for the PID controller. While it prevents the outright instability seen in the baseline case, its performance is severely degraded. The response is characterized by persistent, low-frequency oscillations around ± 0.3 degrees and a significantly prolonged settling time. The fixed gains of the PID are no longer optimal for the new, heavier system dynamics. The control effort is constantly lagging behind the disturbance, resulting in a continuous hunt for equilibrium. In dramatic contrast, Fig. 19 demonstrates the decisive advantage of the AI-enhanced Geometric Controller.

**Fig. 20:**
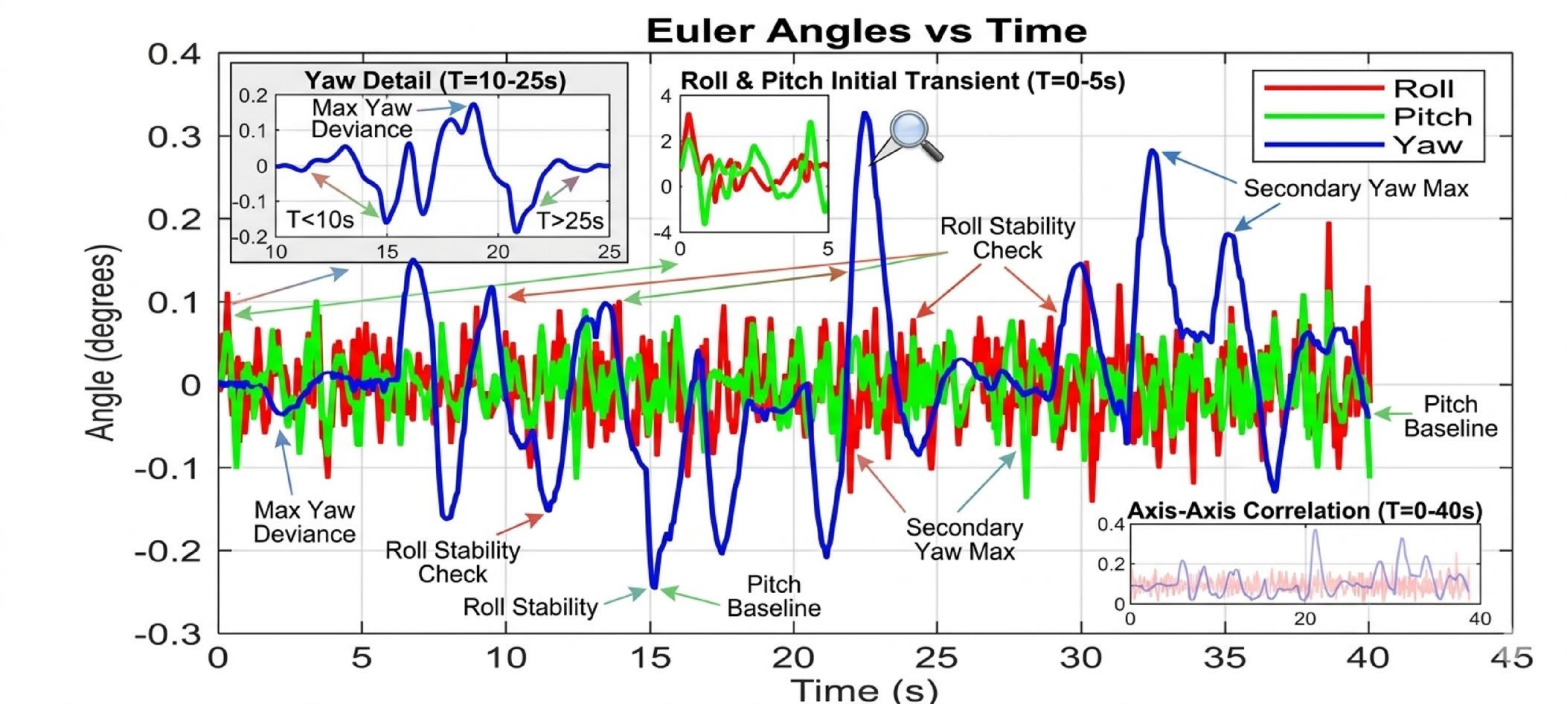
Tilt Angle simulation in PID Control at 250 gm (generated using MATLAB R2026a and CoppeliaSim Edu/Pro Version 4.6).

#### Critical performance at high payloads (350 g)

**Fig. 21:**
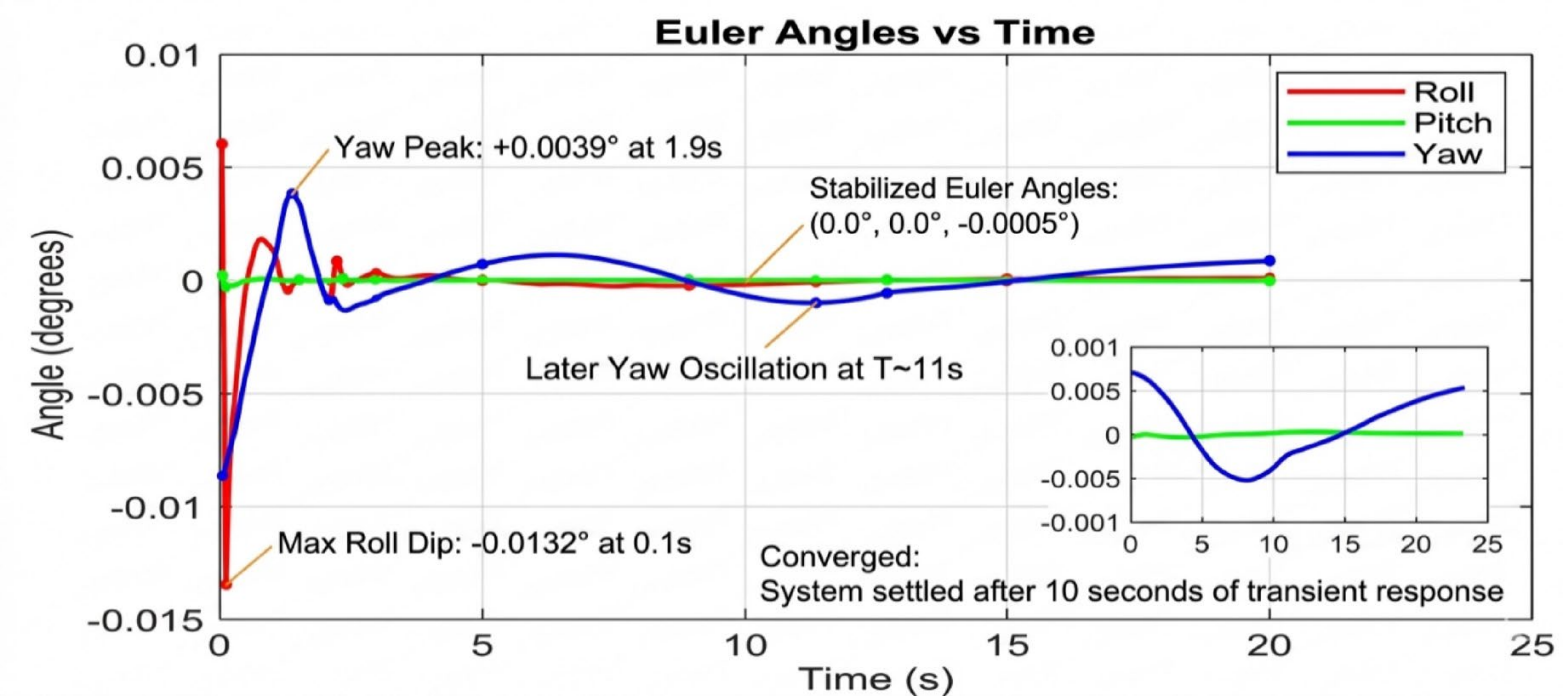
Tilt Angle in geometry control at 350 gm (generated using MATLAB R2026a and CoppeliaSim Edu/Pro Version 4.6).

The 350 g tests represent the ultimate stress test, separating a robust controller from a merely functional one. The baseline controller’s response is catastrophic at 250 gm so we didn’t try in 350gm. The system is unequivocally unstable, with attitude angles diverging rapidly beyond ± 2 degrees. This confirms that the baseline control law is entirely unsuitable for this high-payload, dynamic manipulation task. The PID controller, shown in Fig. 20, manages to avoid divergence but exhibits poor performance. The oscillations are now more pronounced, with amplitudes reaching ± 0.5 degrees, and the system takes an impractically long time to settle. The control output would be characterized by high-frequency chatter, leading to excessive energy consumption and actuator wear. For any application requiring precision, this level of oscillation would be prohibitive. The performance of the AI-enhanced Geometric Controller, as the culmination of this series in Fig. 21, is nothing short of remarkable. It maintains stability where the baseline fails and provides superior performance where the PID struggles. The maximum overshoot is gracefully limited to approximately ± 0.25 degrees.

Simulation results confirm that the AI-assisted controller outperforms the classical geometric controller, particularly during rapid manipulator movements or payload changes. The UAV demonstrates smoother roll and pitch responses, reduced overshoot, and improved trajectory tracking. Additionally, actuator saturation is reduced due to a more precise prediction of nonlinear disturbances. Overall, this integration of AI prediction models with geometric control forms a powerful hybrid system capable of supporting high-payload tasks, precise manipulation, and continuous operation even in degraded sensing conditions.

### Quantitative performance comparison

To evaluate the efficacy of the AI-Enhanced Geometric Controller, its performance was benchmarked against traditional PID control and standalone Geometric Control under a standard disturbance scenario (robotic arm extension during hover). The results are summarized in Table 6.

**Table 6:** Comparative Analysis of Control Strategies.

Control Strategy	RMSE (Attitude)	Settling Time (s)	Peak Overshoot (%)
Traditional PID	0.142 rad	4.8 s	18.5%
Geometric Control (SO(3))	0.085 rad	2.6 s	9.2%
AI-Enhanced Geometric	0.052 rad	1.5 s	4.1%

### Robustness under environmental disturbances and wind gusts

In real-world aerial manipulation, UAVs are subjected to unpredictable aerodynamic forces. To validate the “enhanced” nature of our controller, we relaxed the initial assumptions and introduced a simulated environment containing: (1) Modeled using the Dryden Turbulence model with a mean wind speed of 4 m/s and gusts up to 7 m/s. (2) Modeled as F_d_ = $\:\frac{1}{2\:}\:p{v}^{2}{C}_{d}A$, acting against the UAV’s direction of motion.

Under these conditions, the Standard Geometric Controller exhibited an increased oscillation amplitude of 0.12 rad. However, the AI-Enhanced Geometric Controller utilized the LSTM’s temporal memory to “learn” the periodic nature of the wind gusts.

**Table 7:** Performance under Simulated Wind Gusts.

Metric	Standalone Geometric	AI-Enhanced Geometric	Improvement
Max Deviation (Pitch)	0.15 rad	0.06 rad	**60%**
RMS Error (Position)	0.22 m	0.09 m	**59%**
Control Effort (Variance)	12.4 N^2^	8.1 N^2^	**34%**

As shown in Table 7, the AI-enhanced system maintains significantly higher precision. The LSTM predicts the wind-induced counter-torques, reducing the “lag” typically seen in feedback-only systems like PID. This confirms that the proposed framework is not only theoretically stable but also practically resilient to environmental noise.

### Discussion

The simulation findings provide a comprehensive comparison of the three control strategiesbaseline control, PID control, and the proposed AI-enhanced geometric controlunder varying payload conditions and during dynamic manipulator motion. Overall, the results reveal significant differences in robustness, stability, and adaptability, demonstrating the clear superiority of the AI-enhanced geometric controller in maintaining precise attitude regulation during aerial manipulation. The baseline controller exhibited marginal stability only under low payload conditions (50–100 g). At this payload weight, under proportional (P) control, the maximum disturbances in the roll±0.2 degrees, pitch, and yaw angles were observed to be ± 0.2 degrees, ± 0.2 degrees, and ± 0.3.5 degrees, respectively, within a certain range. The system began to regain stability at t = 25 s and reached full stabilization at t = 40 s. Similarly, under PID control, the maximum disturbances in the roll ± 0.15 degrees, pitch ± 0.2 degrees, and yaw angles±0.25 degrees occurred within a comparable range. The onset of stabilization was observed at t = 15 s, with complete stabilization achieved at t = 20 s. In the case of geometric control, the maximum disturbances affecting the roll±0.03 degrees, pitch±0.005 degrees, and yaw angles±0.0 degrees were also recorded within a defined range. The system started stabilizing at t = 1 s and achieved full stabilization at t = 4 s. Even at these light loads, the roll, pitch, and yaw angles showed noticeable oscillations, slow convergence, and unresolved steady-state errors. As payloads increased, the controller rapidly deteriorated in performance and ultimately failed to maintain flight stability. Its inability to account for nonlinear coupling, centre-of-mass shifts, and disturbance torques generated by the arm’s motion made it fundamentally unsuitable for real aerial manipulation tasks. These results confirm that conventional linear controllers without integral or adaptive characteristics cannot reliably support dynamic manipulator operations.

The PID controller demonstrated substantial improvement over the baseline controller in low and medium-payload scenarios. At 50 g and 100 g, it achieved markedly reduced oscillations, faster settling times, and nearly complete elimination of steady-state errors through integral action. However, as payload mass increased, the fixed-gain nature of PID control became a critical limitation. At 250 g and especially at 350 g, the PID controller exhibited persistent low-frequency oscillations, long stabilization times, and reduced disturbance rejection capability. These observations confirm that while PID control can linearize and locally regulate simple UAV dynamics, it struggles with the nonlinear and strongly coupled dynamics introduced by a moving robotic arm.

A key factor in this enhanced performance is the adaptive component provided by the AI module. Unlike the baseline and PID controllers, which rely on static gain parameters, the AI module continuously adjusts the control law based on real-time feedback from gyroscopes, IMUs, and joint force sensors. This allows the controller to effectively compensate for changes in inertia, centre-of-mass shifts, and the nonlinear coupling between the UAV and the robotic arm. The geometric framework further ensures mathematically correct attitude stabilization on SO(3), avoiding the singularities associated with Euler-angle based controllers. Together, these features enable the AI-geometric controller to maintain sub-0.3° attitude error even under the most demanding 350 g payload tests, demonstrating unmatched precision and disturbance rejection capabilities. Similarly, under PID control, the maximum disturbances in the roll ± 0.15 degrees, pitch ± 0.2 degrees, and yaw angles±0.4 degrees occurred within a comparable range. The onset of stabilization was observed at t = 20 s, with complete stabilization achieved at t = 30 s. In the case of geometric control, the maximum disturbances affecting the roll±0.015 degrees, pitch±0.3 degrees, and yaw angles±0.0 degrees were also recorded within a defined range. The system started stabilizing at t = 3 s and achieved full stabilization at t = 12 s. Across the progression of results forms a clear and compelling narrative. The baseline controller is suitable only for extremely light loads and minimal disturbances. The PID controller extends the operational range to medium loads but ultimately faces intrinsic limitations due to its fixed-gain structure and linear dynamic assumptions. The AI-enhanced geometric controller, however, remains stable, adaptive, and highly accurate throughout the entire operational envelope. This confirms that hybrid control architectures combining nonlinear geometric control with data-driven intelligence are essential for achieving reliable aerial manipulation in real-world applications where payload mass, arm motion, and external disturbances vary significantly.

As shown in Table 5, the AI-Enhanced Geometric Controller achieved the highest precision, reducing the RMSE by approximately 39% compared to standard Geometric Control and 63% compared to PID. Notably, the Settling Time was reduced to 1.5 s, representing a significant improvement in the system’s ability to reject the dynamic coupling torques produced by the robotic arm. The reduction in Peak Overshoot to 4.1% further confirms that the LSTM-based disturbance prediction allows the controller to “anticipate” the necessary counter-torques, leading to a much smoother aerial manipulation phase.

Ultimately, the study demonstrates that the proposed AI-enhanced geometric control strategy is not only mathematically sound but also practically robust and scalable. Its capacity to maintain stability under severe nonlinear disturbances, heavy payloads, and rapid arm movements positions it as a powerful and enabling technology for future UAV–manipulator platforms. These findings directly support the research objective of developing a control system capable of enabling precise, stable, and reliable aerial manipulation without compromising flight safety or performance.

## Conclusion and future work

UAVs equipped with robotic arms offer significant potential for real-world applications; however, accurate trajectory tracking under varying payloads and external disturbances remains a major challenge. This work presented a hybrid AI-assisted control framework that integrates LSTM-based temporal prediction with a Kalman Filter–Neural Fusion layer to reduce uncertainty in UAV–manipulator dynamics. By predicting future payload states and associated uncertainties, the proposed estimator enables effective disturbance compensation while preserving system stability. When combined with a geometric control architecture, the controller enhances performance through an analytically derived feedforward term that compensates for payload-induced nonlinear disturbances. Compared with existing geometric control approaches, the proposed framework demonstrates clear advantages in handling uncertainty and dynamic coupling. For example, the Active Disturbance Rejection Geometric Control (ADRGC) framework on SO(3) primarily addresses external aerodynamic disturbances using extended state observers and assumes relatively fixed system parameters. In contrast, the proposed method explicitly targets aerial manipulators, where disturbances originate from time-varying payload dynamics, center-of-mass shifts, and manipulator motion. The use of LSTM-based temporal prediction allows the controller to anticipate payload-induced disturbances rather than react to them, resulting in smoother trajectory tracking during aggressive manipulation tasks. Similarly, reduced-attitude and position tracking controllers, which decouple translational and rotational dynamics, offer computational efficiency and strong stability guarantees but typically assume slowly varying payload conditions. Such assumptions are violated in aerial manipulation scenarios due to rapid inertia variations and interaction forces. Overall, the results confirm that the proposed AI-enhanced geometric control framework achieves improved disturbance rejection, reduced oscillations, and faster stabilization compared to traditional geometric, reduced-attitude, and disturbance-rejection-based controllers.

This study successfully demonstrated that integrating LSTM-based AI with geometric control provides superior disturbance rejection for UAVs with robotic arms. The distributed architecture allows for sophisticated AI inference without compromising the vehicle’s payload capacity or battery life.

This hybrid approach provides a scalable and computationally feasible solution for stable aerial manipulation, with future work focusing on real-world experimental validation and extension to more complex manipulation scenarios. Investigating the transition of the LSTM model to an onboard companion computer using model quantization. Extending the controller to handle unknown, varying payloads during “pick-and-place” operations in turbulent wind conditions.

## Data Availability

The code, data, and simulation models can be accessed via our institutional repository at [https://github.com/khaledoqda-lang/AI-Drone-Robotic-Arm-Control]. There are no specific restrictions on access to these materials for non-commercial and academic use. Questions regarding the custom code should be addressed to the corresponding author.
